# Fibroblast activation in response to TGFβ1 is modulated by co-culture with endothelial cells in a vascular organ-on-chip platform

**DOI:** 10.3389/fmolb.2023.1160851

**Published:** 2023-07-28

**Authors:** Rebeccah J. Luu, B. Christopher Hoefler, Ashley L. Gard, Casey R. Ritenour, Miles T. Rogers, Ernest S. Kim, Jonathan R. Coppeta, Brian P. Cain, Brett C. Isenberg, Hesham Azizgolshani, Oscar R. Fajardo-Ramirez, Guillermo García-Cardeña, Matthew P. Lech, Lindsay Tomlinson, Joseph L. Charest, Corin Williams

**Affiliations:** ^1^ Bioengineering Division, The Charles Stark Draper Laboratory Inc., Cambridge, MA, United States; ^2^ Pfizer Inc., Groton, CT, United States; ^3^ Laboratory for Systems Mechanobiology, Center for Excellence in Vascular Biology, Department of Pathology, Brigham and Women’s Hospital, Harvard Medical School, Boston, MA, United States; ^4^ Pfizer Inc., Cambridge, MA, United States

**Keywords:** co-culture, organ-on-chip, vascular, transforming growth factor β1, myofibroblast, fluid shear stress, fibrosis

## Abstract

**Background:** Tissue fibrosis is a major healthcare burden that affects various organs in the body for which no effective treatments exist. An underlying, emerging theme across organs and tissue types at early stages of fibrosis is the activation of pericytes and/or fibroblasts in the perivascular space. In hepatic tissue, it is well known that liver sinusoidal endothelial cells (EC) help maintain the quiescence of stellate cells, but whether this phenomenon holds true for other endothelial and perivascular cell types is not well studied.

**Methods:** The goal of this work was to develop an organ-on-chip microvascular model to study the effect of EC co-culture on the activation of perivascular cells perturbed by the pro-fibrotic factor TGFβ1. A high-throughput microfluidic platform, PREDICT96, that was capable of imparting physiologically relevant fluid shear stress on the cultured endothelium was utilized.

**Results:** We first studied the activation response of several perivascular cell types and selected a cell source, human dermal fibroblasts, that exhibited medium-level activation in response to TGFβ1. We also demonstrated that the PREDICT96 high flow pump triggered changes in select shear-responsive factors in human EC. We then found that the activation response of fibroblasts was significantly blunted in co-culture with EC compared to fibroblast mono-cultures. Subsequent studies with conditioned media demonstrated that EC-secreted factors play at least a partial role in suppressing the activation response. A Luminex panel and single cell RNA-sequencing study provided additional insight into potential EC-derived factors that could influence fibroblast activation.

**Conclusion:** Overall, our findings showed that EC can reduce myofibroblast activation of perivascular cells in response to TGFβ1. Further exploration of EC-derived factors as potential therapeutic targets in fibrosis is warranted.

## Introduction

Tissue fibrosis is a global healthcare burden which has no effective treatments for either prevention or resolution of the fibrotic lesions (Henderson et al., 2020). Fibrosis can occur in various tissues such as the heart (Frangogiannis, 2020), lung (Martinez et al., 2017), liver (Hernandez-Gea and Friedman, 2011), and kidney (Duffield, 2014), leading to organ failure, the need for organ transplant, or, if left untreated, death. It is postulated that fibrosis is responsible for up to 45% of deaths in the industrialized world (Henderson et al., 2020). Current research efforts in fibrosis focus on various aspects of fibrotic progression, from the influence of circulating and resident immune cells (Huang et al., 2020), to alterations in the extracellular matrix (ECM) (Herrera et al., 2018), to the organ-specific cells that initiate the fibrotic lesion in the perivascular space: pericytes (Schrimpf and Duffield, 2011) and fibroblasts (Kendall and Feghali-Bostwick, 2014). Perivascular cells in healthy organs are involved in tissue support and normal wound healing (Bainbridge, 2013); however in a fibrotic disease context they undergo a myofibroblast transition and produce excessive ECM (Greenhalgh et al., 2013). While numerous anti-fibrotic approaches have been attempted, from disrupting the inflammatory pathways that lead to upregulation of TGFβ1 to targeting the collagen that has been deposited by the activated cells (e.g., LOXL2), no significant clinical successes have yet been achieved (Zhao et al., 2022). Given the complexity of the fibrotic milieu, a better understanding of perivascular cell interactions with their local microenvironment is paramount to finding an effective treatment for tissue fibrosis.

Across many fibrotic diseases, the central role of transforming growth factor beta 1 (TGFβ1) in the pro-fibrotic activation of the ECM-producing cells has been noted (Kim et al., 2018). Specifically, studies have shown that TGFβ1 is a strong activator of perivascular cells such as hepatic stellate cells (Dewidar et al., 2019), renal fibroblasts and pericytes (Lin et al., 2008), and lung fibroblasts and pericytes (Hung et al., 2013). Similarly, in both preclinical animal models and in human disease samples, the TGFβ1 pathway is upregulated relative to healthy controls (Ramachandran et al., 2019; 2020). In an *in vitro* setting, TGFβ1 activation is characterized by key transcriptional and proteomic changes, but among the most notable hallmarks are the *de novo* expression of α-smooth muscle actin (SMA) expression and deposition of collagen I (Abdalla et al., 2013; Hinz, 2016).

While the role of pericytes and fibroblasts in fibrogenesis is well accepted, it is crucial to consider the influence of other cell types in modulating the activation response. Numerous studies and reviews to date have focused on pericyte/fibroblast interactions with organ-specific epithelial cells (Sakai and Tager, 2013; Wu et al., 2013; Prestigiacomo et al., 2017) and immune cells (O’Reilly et al., 2012; Pincha et al., 2018; Carter and Friedman, 2022). However, given the proximity of fibroblasts and pericytes to microvascular endothelial cells (EC) within these organs, their interactions with EC should also be considered. It is well-known that EC cross-talk with other cell types is important for the health of various organs, such as the heart (Colliva et al., 2020). Of particular relevance, liver sinusoidal EC are known to maintain the quiescence of hepatic stellate cells (DeLeve et al., 2008; Poisson et al., 2017). Notably, fibrosis tends to initiate around the microvasculature (Lin et al., 2008; Ytrehus et al., 2018) and aberrant angiogenesis is an early event in fibrosis (Park et al., 2015; Liu et al., 2017). Taken together, these phenomena point to the potential role of EC in modulating early fibrotic signaling and events.

Although animal models have yielded invaluable insights into fibrosis (Padmanabhan et al., 2019), the systematic study of cell-cell interactions and cellular mechanisms *in vivo* is challenging. Simplified culture systems lack control over cell interactions and/or important biophysical cues such as fluid flow. Complex *in vitro* culture platforms such as microphysiological systems or organ-on-chip models can help address these challenges by providing precise spatial and/or temporal control over variables of interest, including specific cell types, ECM components, fluid flow, and pro-fibrotic perturbations. Complex *in vitro* systems also offer promise for drug discovery and testing, such as models of the human liver for interrogating nonalcoholic steatohepatitis (Feaver et al., 2016) and human cardiac fibrosis-on-chip (Mastikhina et al., 2020). While some complex models include EC, their role in fibrotic responses is generally not studied.

Fluid flow, and in particular, fluid shear stress (FSS), becomes a key variable when considering models that include vascular endothelium. FSS is a critical regulator of EC health and disease, and can influence EC morphology (Malek and Izumo, 1996), proliferation (Kadohama et al., 2007), phenotype (Topper and Jr, 1999), barrier function (Buchanan et al., 2014), gene expression (Braddock et al., 1998), and secreted factors (Galbusera et al., 1997; Yu et al., 2002; Dardik et al., 2005; Chung et al., 2022). Interestingly, a few studies have investigated the role of FSS in the activation responses of fibroblasts and stellate cells (Lei et al., 2020; Swain et al., 2022), but not EC. A model of EC-perivascular cell interactions in the presence of pro-fibrotic perturbations should include physiologically relevant FSS applied to the endothelial layer to better mimic *in vivo* conditions.

**FIGURE 1:**
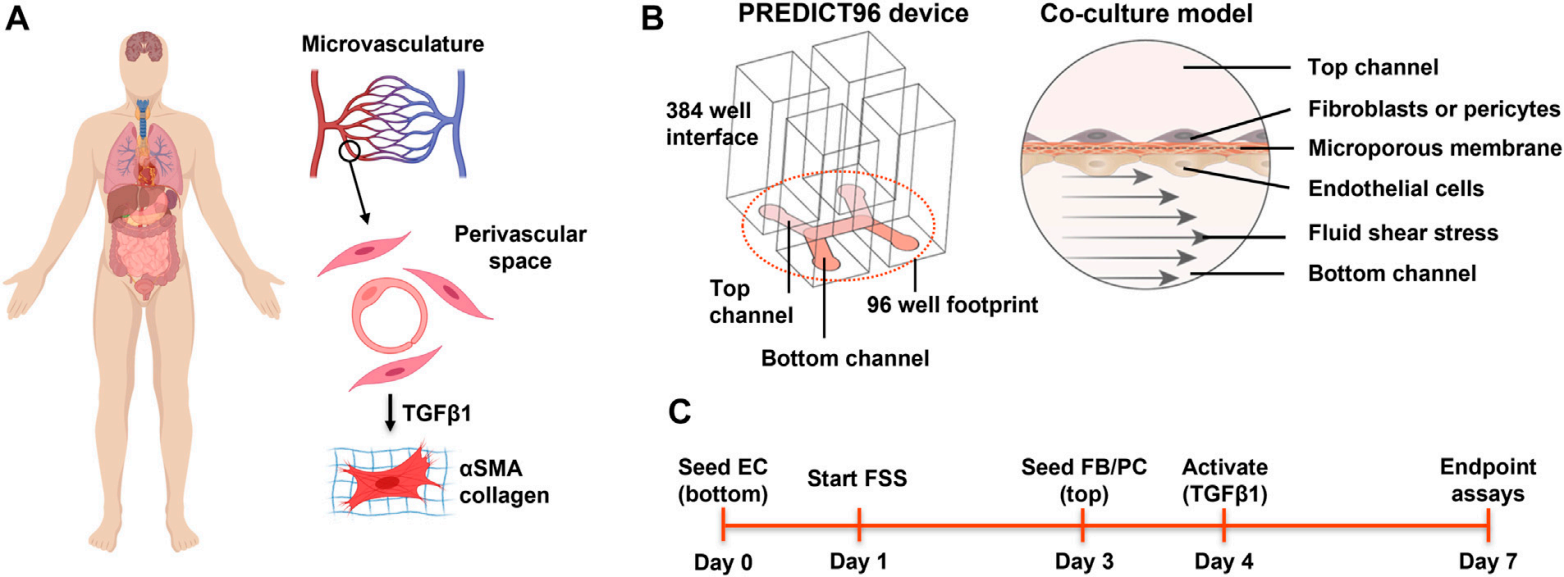
Overview of the perivascular fibrosis/activation model. **(A)** Many soft tissues and organs are subject to fibrosis. Activation of pericytes and fibroblasts to a myofibroblast phenotype plays a key role in pathogenesis across tissue types. Given the proximity of these cell types to capillaries, how might endothelial cells influence the response to pro-fibrotic factors? **(B)** Schematic of single PREDICT96 device and the endothelial-perivascular cell co-culture model. **(C)** Overview of the experimental timeline used. EC = endothelial cell; FB = fibroblast; PC = pericyte; FSS = fluid shear stress. Panel A created with BioRender.com.

The goal of this work was to investigate the influence of EC on the response of perivascular cells to the pro-fibrotic factor TGFβ1 (Figure 1). Our approach was to use a high throughput organ-on-chip platform, PREDICT96 (Azizgolshani et al., 2021), consisting of 1) a plate containing 96 bilayer microfluidic devices ideal for studying co-culture interactions (Rogers et al., 2021) and 2) a high flow pump lid that can achieve a range of fluid shear stress (FSS) levels that are relevant for human vascular endothelium (Hathcock, 2006). We first studied the response of lung fibroblasts, dermal fibroblasts, and retinal pericytes to TGFβ1 and found that these different perivascular cell sources had varying sensitivity to TGFβ1 activation as measured by SMA expression. We then validated the shear-responsiveness of human EC in the PREDICT96 system when subjected to “low” (0.5 dyn/cm^2^) and “high” (7 dyn/cm^2^) physiological FSS conditions. Next, we investigated the TGFβ1 activation response of fibroblasts when co-cultured with EC. Interestingly, co-culture with EC induced low level basal activation in a fraction of fibroblasts, but exogenous TGFβ1 stimulation resulted in significantly lower activation in co-cultured fibroblasts compared to mono-cultures. Conditioned media from ECs also blunted activation of fibroblast mono-cultures, suggesting that the effect is at least partially mediated by EC-secreted factors. A Luminex panel and single cell RNA-sequencing (scRNA-seq) study yielded additional insight into potential factors that are differentially expressed in the EC that could affect myofibroblast activation response. Overall, these insights warrant deeper investigation in future studies to identify novel EC-derived factors as potential therapeutic targets in fibrosis.

## Materials and methods

### Fabrication of PREDICT96 plates and high flow pump

The PREDICT96 plates and the high flow pump were fabricated in-house at Draper as previously described (Azizgolshani et al., 2021). The PREDICT96 microfluidic culture plates consisted of 96 arrayed bilayer devices with channels of 240 µm depth separated by a 20 µm thick microporous polycarbonate membrane (AR Brown) with 1 µm diameter pores. This pore size was chosen to facilitate cell-cell communication while preventing cell migration across the membrane. The high flow pump consisted of a lid containing 96 individual micro-pumps. The pump lid had 2-region control such that the left 48 and right 48 pumps could run at different flow rates. The pump was calibrated as described previously (Azizgolshani et al., 2021) to determine the average stroke volume across the 96 micro-pumps, and used to set the desired flow rates for the experiments.

### Cell culture

Vendor and donor details for the cell types used in this work are provided in Table 1. All cells were expanded according to their respective manufacturer’s protocols to establish baseline frozen stocks; this ensured consistent procedures and passage numbers across experiments. Primary human retinal microvascular EC were purchased from Angio-Proteomie (Boston, MA) and expanded in the manufacturer’s Endothelial Growth Medium. This EC source was used for all co-culture studies conducted. Primary human dermal fibroblasts and normal human lung fibroblasts were purchased from Lonza (Basel, Switzerland) and expanded in the manufacturer’s recommended medium, Fibroblast Growth Medium-2 (FGM-2, catalog # CC-3132; comprised of FBM™ Basal Medium and FGM™-2 SingleQuots™ supplements). Immortalized human retinal pericytes were generated by Pfizer from primary cells acquired from Angio-Proteomie and expanded in Angio-Proteomie’s Pericyte Growth Medium.

**TABLE 1:** Cell source information.

Cell type	Vendor	Catalog #	Donor Characteristics	Passage # used
Retinal MVECs	Angio-Proteomie	cAP-0010	Female, 25 years old	P7
Retinal Pericytes	Angio-Proteomie	cAP-0025	hTERT-immortalized from primary source	P6
hDF	Lonza	CC-2509	Male, neonatal	P6
NHLF	Lonza	CC-2512	Male, 12 years old	P6

For the experiments described in this study, cells were thawed into flasks with either EGM-2MV (Lonza, catalog # CC-3202) for EC or FGM-2 for fibroblasts and pericytes. Media was changed the next day. Cells typically reached 70%–80% confluence within 2–4 days and were harvested for seeding in PREDICT96 plates as described below.

### Cell seeding in PREDICT96

Immediately prior to cell seeding, PREDICT96 plates were plasma treated for 120 s to render the device channels hydrophilic. Devices were then washed with 70% ethanol for 5 min, distilled water twice, and phosphate buffered saline (PBS) once, followed by coating with 5 μg/mL of fibronectin (Millipore Sigma) for 2 h at 37°C. Afterward, the plates were primed with MCDB131 Complete Media (composed of MCDB131 base media, 1x Microvascular Growth Supplement, 1x Gibco GlutaMAX Supplement, and 1x penicillin-streptomycin, all purchased from Thermo Fisher Scientific). EC were seeded into the bottom channels at 1.8 × 10^6^ cells/mL with an inlet-outlet volume differential of 35–15 µL. The volume differential was used to ensure flow-through of cells into the device channels. Once the port volumes equilibrated (∼2 min), the plates were flipped upside down to allow the cells to settle on the underside of the membrane. The plates were incubated in this configuration for 2 h at 37°C. Afterward, the plates were carefully flipped right side up, the media was aspirated and replenished with fresh MCDB131 Complete, and the PREDICT96 plates were placed in the incubator overnight. FSS was applied to the bottom channels the next day, as described in the sub-section below.

Three days after EC seeding, fibroblasts were seeded into the top channel at 250,000 cells/mL in Co-Culture Media composed of MCDB131 base media supplemented with 0.5% fetal bovine serum, 1x Chemically Defined Lipid Concentrate (Thermo Fisher Scientific), 1x Insulin-Transferrin-Selenium (Thermo Fisher Scientific), 1x Gibco GlutaMAX Supplement (Thermo Fisher Scientific), 1x Penicillin-Streptomycin (Thermo Fisher Scientific), and 100 µM ascorbic acid (2-O-a-D-Glucopyranosyl-L-ascorbic acid, Sigma).

Note that for mono-culture conditions, the cells were seeded in the same channels as would have been used in co-culture models: fibroblasts in the top channel of devices, EC in the bottom channel.

### Application of fluid shear stress

The two-region high flow pump was sterilized with ethylene oxide and then degassed in a vacuum chamber for at least 1 week prior to use. The pump was removed from the sterilization bag in a biosafety cabinet and pneumatic connectors were set up as previously described (Azizgolshani et al., 2021). The pump was primed by pumping for 5 min in a reservoir containing 70% ethanol, followed by twice with distilled water. Prior to putting the pump on the PREDICT96 plate, the pumps were primed in culture media. At 24 h after seeding the EC, FSS was applied to the EC-containing channel. A first order approximation of FSS in a rectangular channel was calculated, with channel depth of 240 μm, channel length of 7.6 mm, and media viscosity of 0.653 mPa*s. Low FSS was initiated at 0.5 dyn/cm^2^ (48 μL/min) in one-half of the PREDICT96 plate while high FSS was set at 7 dyn/cm^2^ (600 μL/min) in the other half. Intermediate FSS were also used for a sub-set of experiments described below: 2 dyn/cm^2^ (175 μL/min) and 4 dyn/cm^2^ (350 μL/min).

### Validation of endothelial cell response to fluid shear stress

Human EC response to FSS applied with the PREDICT96 vascular pump was validated using a KF2 reporter cell line (Slegtenhorst et al., 2018) and measurement of the expression of shear-responsive genes, KLF4 and PECAM1/CD31 (Fleming et al., 2005; Tzima et al., 2005; Hamik et al., 2007; Fan et al., 2017; Xie et al., 2020) in the retinal microvascular EC described above. KLF2 reporter cells were generated as previously described (Slegtenhorst et al., 2018). Briefly, human umbilical vein endothelial cells were infected with a lentivirus encoding the KLF2-GFP construct, and then were sorted based on the level of GFP expression. A homogenous GFP-expressing population (GFP^low^) was sorted by exclusion of GFP negative cells (GFP^−^) and the brightest expressers (GFP^high^). These KLF2 reporter cells were seeded into PREDICT96 devices as described above and cultured in Lonza EGM-2MV media for the duration of the experiment. FSS was applied for 48 h at 0.5 and 7.5 dyn/cm^2^ in the left and right halves of the plate, respectively. The cell nuclei were stained with Hoechst 33342 and then a sub-set of devices were imaged immediately on a Zeiss LSM700 confocal microscope with Zen Black software to assess KLF2 expression. FSS was then resumed for another 48 h at 2 and 4 dyn/cm^2^ and imaged. A near static, feeder flow condition (10 μL/min) was imaged at the beginning and end of the experiment to calibrate baseline fluorescence. A FSS “dose response” curve was then generated from the percentage of KLF2+ reporter cells across conditions. The KLF2 reporter cells were only used for this FSS response validation study. KLF4 and PECAM1 gene expression were determined for retinal microvascular EC subjected to low and high physiological FSS according to RT-qPCR methods described below. In addition, we examined the ultrastructure of these EC subjected to low and high FSS via transmission electron microscopy, as described below.

### Activation with TGFβ1

Fibroblasts and pericytes in mono-culture or co-culture were activated with recombinant human TGFβ1 (R&D Systems) at 24 h post-seeding. Dose response curves were determined for mono-cultures of normal human lung fibroblasts (NHLFs), human dermal fibroblasts (hDFs), retinal pericytes (RPC), and retinal microvascular EC. For co-culture studies of ECs and fibroblasts (hDF), 10 ng/mL TGFβ1 was used. In all cases, TGFβ1 was added at a single time point to the top channels of devices containing the pericyte or fibroblast cell type and incubated for 72 h. TGFβ1 stock solution was made fresh for each experiment in a vehicle solution containing 0.5% bovine serum albumin (BSA, Sigma) and 4 mM HCl (Sigma). The high flow pump was stopped for less than 30 min for dosing.

### Immunocytochemistry and imaging

At 72 h post-activation, PREDICT96 devices or mono-cultures in 96 well plates were fixed with cold 95% methanol/5% acetic acid (vol/vol) for 12 min at 4°C. After washing three times with PBS, the samples were blocked with 3% normal goat serum (NGS, Thermo Fisher) for 1 h at room temperature (RT). Primary antibodies were then added in 3% NGS and incubated for 2 h at RT, as follows: ERG1 at 1:250 (rabbit polyclonal, Abcam) or PECAM1 at 1:150 (rabbit polyclonal, Abcam) to label EC and SMA at 1:250 dilution (mouse monoclonal, 1A4, Sigma) to label activated myofibroblasts. The samples were washed three times with PBS for at least 5 min each and then incubated in secondary antibody solution in 3% NGS for 1 h at RT, as follows: Alexa Fluor 488 goat anti-rabbit IgG at 1:250 (Thermo Fisher), Alexa Fluor 568 goat anti-mouse IgG at 1:250, and Hoechst 33342 at 4 μg/mL (Thermo Fisher). After washing three times with PBS, the stained plates were stored wrapped in foil at 4°C until imaging.

Images of PREDICT96 devices or wells were captured with a Zeiss LSM700 confocal microscope and Zen Black 2012 software (version 14.0.17.201). Imaging parameters were adjusted manually for each color channel and then held constant across the experiment. Tile scans of PREDICT96 channel overlap area or 96 wells were acquired with a ×10 objective lens and 0.5 digital zoom. Raw images were analyzed in ImageJ Fiji to measure mean fluorescence intensity (MFI) of SMA or collagen. Normalization of MFI for each experiment is described in the respective figure captions, where a maximum responder or designated control was assumed to be 100% activation. The rationale for this method of analysis was to provide a standard normalization approach to allow for relative comparisons across conditions and experiments, as absolute values of MFI can fluctuate experiment to experiment due to variations in biological or technical parameters.

### Reverse transcription quantitative polymerase chain reaction

Cells in devices were washed twice with PBS and then detached by treating with Accutase for 5 min. Devices were then washed twice with media and the samples were collected into 1.5 mL microcentrifuge tubes, noting that cells from each device channel were collected in separate tubes to analyze the EC and fibroblast populations separately. Cells were spun down at 2000 rpm and the supernatant was removed. The cell pellets were lysed with RLT lysis buffer (Qiagen, cat # 79216) and stored at −80°C until further processing. Samples were processed using RNEasy Micro Kits (Qiagen, cat # 74004). cDNA was made using SuperScript IV Vilo Mix (Thermo Fisher). Reverse transcription quantitative polymerase chain reaction (RT-qPCR) was performed using Taqman Gene Expression Assays (Thermo Fisher, Table 2) and Taqman Fast Advanced Master Mix (Thermo Fisher, cat # 4444557). The reaction was run on an Applied Biosystems QuantStudio 7 Flex System (Thermo Scientific). Transcript expression was quantified by using the method described by Schmittgen and Livak (Livak and Schmittgen, 2001), utilizing the Comparative cycle threshold (Ct) values and GAPDH as the reference gene. Samples extracted from *n* = 4 devices per condition were tested in technical duplicates. Ct values of samples from the same condition were averaged during analysis. Details on the genes of interest are provided in Table 2.

**TABLE 2:** Genes analyzed.

Gene/Probe	Catalog number, thermo Fisher	Purpose/Function
ACTA2	Hs00426835_g1	TGFβ1 activation
COL1A1	Hs00164004_m1	TGFβ1 activation
TGFBR1	Hs00610319_m1	TGFβ1 activation
TGFBR2	Hs00559661_m1	TGFβ1 activation
KLF4	Hs00358836_m1	EC, shear responsive
PECAM1	Hs01065279_m1	EC, shear responsive
GAPDH	Hs02786624_g1	Housekeeping

### Immunogold labeling and transmission electron microscopy

To examine cellular ultrastructure in the devices, transmission electron microscopy was used. The experiment included EC cultured under low and high FSS. At the conclusion of the experiment (7 days under FSS), all labeling methods were carried out over ice (0–4^O^C). The media was aspirated from the channels of the devices and the cells were washed with PBS. Nonspecific labeling was blocked by incubating the cells with non-related antibody (Aurion Goat Gold Conjugate) in PBS for 30 min. Cells were washed with fresh PBS/BSA-concentrate prior to incubation with the primary antibody (anti-CD31 antibody diluted at 1:10, 1:50, 1:100) for 2 h. Washing steps with PBS/BSA-c followed the primary antibody incubation and preceded incubation with the secondary immunogold labeled antibody (Goat anti-rabbit IgG 15 nM, diluted 1:10) for 2 h. Additional washes preceded fixation with Karnovsky’s fixative containing 0.1 M phosphate-buffer, 2% paraformaldehyde and 2.5% glutaraldehyde. Cells in channels were fixed for 2 h then transferred to 0.1 M Sorensen’s phosphate buffer and refrigerated overnight.

All devices were post-fixed in 0.1 M phosphate-buffered 1% osmium tetroxide, dehydrated in graded ethanol series, and embedded in epoxy resin. Select channels from the PREDICT96 plate were manually extracted using a blade. Prepared tissue blocks were trimmed and semi-thin (0.6 mm) sections were prepared from select specimens, mounted to glass slides, stained with 1% toluidine blue, and examined via light microscopy. These semi-thin sections were used to locate the area of the membrane and cell layers of interest, and selected blocks were further trimmed to these sub-regions. Thin sections (∼90 nm) of the sub-regions were prepared, stained, and examined using a Hitachi H 7100 transmission electron microscope. Digital micrographs (Advanced Microscopy Techniques, Corp.,) of representative areas were collected.

### Conditioned media experiments

In some experiments, conditioned media were added simultaneously with TGFβ1 to assess the potential inhibitory effects of cell-secreted factors. For these studies, EC mono-cultures, EC and hDF co-cultures, and hDF mono-cultures were established in PREDICT96 devices for 72 h under high FSS. The media was then collected from respective cell type channels and pooled for conditioned media stocks (EC mono-culture, EC co-culture, hDF mono-culture, hDF co-culture). The collected conditioned media were used immediately on hDF mono-cultures in 96 well plates. The hDF mono-cultures, seeded at 7,500 cells per well in co-culture media, were treated with conditioned media with or without TGFβ1 at 10 ng/mL for 72 h. Control conditions were hDF mono-cultures treated with non-conditioned co-culture media, with or without TGFβ1. After 72 h, a fluorescent collagen probe was added to each condition in fresh media and incubated for an additional 24 h before fixing and staining for SMA and Hoechst.

### Fluorescent collagen probe

In hDF mono-culture experiments, a live fluorescent collagen probe was used to label mature collagen synthesized by the cells. CNA35 probes have high affinity and selectivity for collagen (Krahn et al., 2006; Aper et al., 2014), and were created according to previously published methods (Tan et al., 2019), and described briefly here. The pET28a-EGFP-CNA35 vector was a gift from Maarten Merkx (Addgene plasmid #61603; http://n2t.net/addgene:61603; RRID:Addgene_61603). This vector contains the CNA35 collagen probe, created by fusing the collagen binding domain of *Staphylococcus aureus* collagen binding adhesin CNA with GFP. The vector was transformed into BL21 (DE3) *E. coli*. Protein production was induced with 1 mM Isopropyl ß-D-1-thiogalactopyranoside (IPTG) after which cultures were grown overnight with shaking at 37°C. Cells were recovered by centrifugation and resuspended in 20 mM Tris-HCl (pH 7.9), 0.5 M NaCl, 30 mM imidazole. Cells were lysed using an LM-10 microfluidizer (Microfluidics International Corp, Westwood, MA) and purified using Ni-NTA agarose (ThermoFisher, catalog # 88222), pre-equilibrated and washed with resuspension buffer. Protein was eluted using 0.5 M imidazole and dialyzed in 50 mM Tris-HCl (pH 8.0), 100 mM NaCl. Purification and dialysis were shielded from light exposure using aluminum foil. After purification, samples were incubated in the dark at 37°C to allow complete chromophore maturation. Proteins were quantified and diluted to 10 µM final concentration. Aliquots were stored at −80°C and thawed on ice immediately prior to use. The probe was added to the cultures at a dilution of 1:50 and allowed to bind overnight. Samples were then fixed and stained according to the methods described above.

### Luminex panel for secreted factors

In order to identify potential secreted factors of interest from EC and hDF, media was collected from the top and bottom channels of individual PREDICT96 devices and immediately frozen at −80°C until use. Cytokine secretion was quantified using a Human XL Cytokine Magnetic Luminex Performance Assay 45-plex Fixed Panel (R&D Systems, catalog # LKTM014). The samples were processed by following the manufacturer’s protocol and analyzed with a Luminex FLEXMAP 3D System. The data collected were used to generate standard curves for each analyte using the manufacturer’s specified point curve-fit for each analyte. The concentration of the secreted factors was determined for each individual PREDICT96 device channel. Devices derived from the same experimental conditions were averaged (*n* = 4). Samples that were below the limit of detection of the kit were removed from the averaged analysis, but included on the graphs for comparison (represented by triangles).

### Cytokine and growth factor treatments

For activation studies involving individual factors identified in EC channels by Luminex, hDF mono-cultures were seeded into 96 well plates as described above. At 3 h post-seeding, the media was refreshed and dosed with respective factors of interest to simulate exposure to these factors during co-culture. All factors were reconstituted into stock solutions according to the manufacturer’s instructions and used at 1 or 10 ng/mL. At 24 h post-seeding, the media was refreshed with individual factors plus TGFβ1 at 10 ng/mL. After 72 h, the media was refreshed with the fluorescent collagen probe added. The next day, the samples were fixed and stained for SMA and Hoechst. Details on the growth factors and cytokines used are provided in Supplementary Table S1.

### Single cell RNA sequencing

Single cell RNA sequencing (scRNA-seq) was run on a set of control and TGFβ1-treated samples subjected to high FSS. EC mono-cultures, hDF mono-cultures, and EC-hDF co-cultures were extracted from devices using Accutase and checked for cell count and viability by flow cytometry using acridine orange and propidium iodide. One device per condition was also stained for CD31 and SMA to ensure the models responded as expected.

Single cell RNA sequencing libraries were prepared from >90% live EC and hDF, freshly isolated from PREDICT96 devices using a 10X Chromium 3’ reagent kit (v3.1). Target recovery was 10,000 cells. The final library was sequenced on a NovaSeq SP flow cell with a yield of 772M 150 bp reads. After pseudoalignment (Melsted et al., 2019) to the GRCh38 reference genome, 72% of the reads were retained (561M aligned reads, ∼50 k per cell average), and ∼10,000 non-empty droplets were identified (inflection point of UMI distribution plot). After removing empty droplets, averages of ∼28,000 UMIs and ∼5,000 genes were detected per cell. Low-quality cells were filtered using relatively stringent criteria. Cells with <2,000 genes detected and a >20% fraction of mitochondrial gene UMIs detected were removed prior to further analysis.

For cell clustering, the top 20% most variable genes were identified using scran (Lun et al., 2016). Principal components (10 dims) and UMAP projections (2 dims) (McInnes et al., 2018) were constructed using monocle3 (Cao et al., 2019). Cells were clustered using the Leiden community detection (Traag et al., 2019) method at a resolution of 0.001. Marker genes for each cluster were identified in two passes, first by a Jensen-Shannon specificity score, followed by a likelihood ratio test against a reference sample of cells, both implemented in the top_markers function of monocle3. To specifically look at how gene expression changed between clusters of cells, sets of genes with spatially correlated patterns of expression were identified using graph autocorrelation analysis (implemented in monocle3 by functions graph_test and find_gene_modules). Genes with highly localized expression patterns within clusters and abrupt changes in expression between clusters were selected for further analysis. Gene enrichment analysis was performed using the GSEA method (Subramanian et al., 2005) with the hallmark and transcription factor target gene sets from MSigDB (Liberzon et al., 2015). Genes identified previously were grouped by enriched terms, and average gene expression for each term was calculated for each cluster. Heatmaps of average log counts of each term within each cluster were plotted using pheatmap.

### Statistical analysis

Statistical analyses were performed in GraphPad Prism (version 9.3.1). Ordinary one- or two-way analysis of variance (ANOVA) with Tukey’s or Sidak’s *post hoc* tests were used where appropriate and are noted in the figure legends. For the Luminex panel, multiple unpaired t-tests were used to compare EC vs hDF channel-specific factors. Note that due to the complexity of the statistics, only those most relevant to the results and discussion are indicated on the graphs for *p*-values <0.05. In addition, we determined intra- and inter-plate variability by calculating the coefficient of variance for SMA MFI across four conditions (hDF mono-culture without TGFβ1, hDF mono-culture with TGFβ1, hDF co-culture without TGFβ1, hDF mono-culture with TGFβ1) within a single PREDICT96 plate and across 3 independent PREDICT96 plates.

## Results

### Different perivascular cell sources demonstrate variable sensitivity to TGFβ activation

**FIGURE 2:**
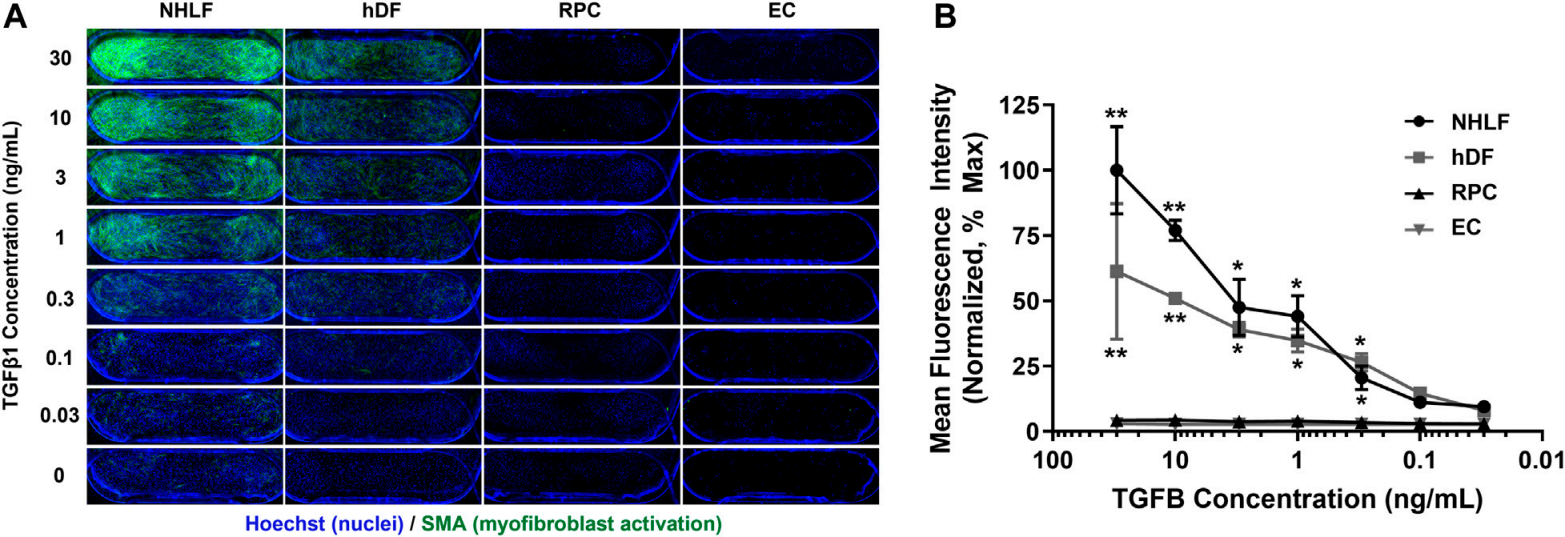
Dose response of different perivascular cell types and EC to TGFβ1 stimulation in mono-culture. **(A)** Representative images of cells stained for Hoechst 33342 (blue) and SMA (green). Images show PREDICT96 device channel overlap area (3 mm × 1 mm). **(B)** Quantification of SMA mean fluorescence intensity (MFI) normalized to the maximum responder (NHLF at highest TGFβ1 dose of 30 ng/mL, assumed 100% activation) as a function of TGFβ1 concentration. NHLF were the most responsive to TGFβ1; hDF had a mid-level response, and RPC had very low activation in response to TGFβ1. EC did not express SMA. NHLF = normal human lung fibroblast; hDF = human dermal fibroblast; RPC = retinal pericyte; EC = endothelial cell.

Given that multiple organs within the human body can undergo fibrosis, we first studied TGFβ1-induced activation of several perivascular cell types derived from different human tissues. Dose response curves were generated for 2 fibroblast types (NHLF and hDF), pericytes (RPCs), and primary human retinal microvascular endothelial cells (EC). We chose retinal microvascular EC as the EC source for our model due to their high blood EC population (Nakao et al., 2012) compared to microvascular EC sourced from other organs which often contain lymphatic EC (Kriehuber et al., 2001). We found that different donors and organ sources of perivascular cells had varying sensitivities to TGFβ1, but all responded with increased SMA expression in a dose-dependent manner (Figure 2). NHLF had the highest activation as measured by SMA expression, followed by hDF. RPC had very low response to TGFβ1 compared to NHLF and hDF. The EC source used for these experiments did not express SMA even at the highest dose of TGFβ1 tested. For the remainder of the studies, we focused on the medium-responsive hDF as the perivascular cell type.

### Development and validation of endothelial cells under fluid shear stress

**FIGURE 3:**
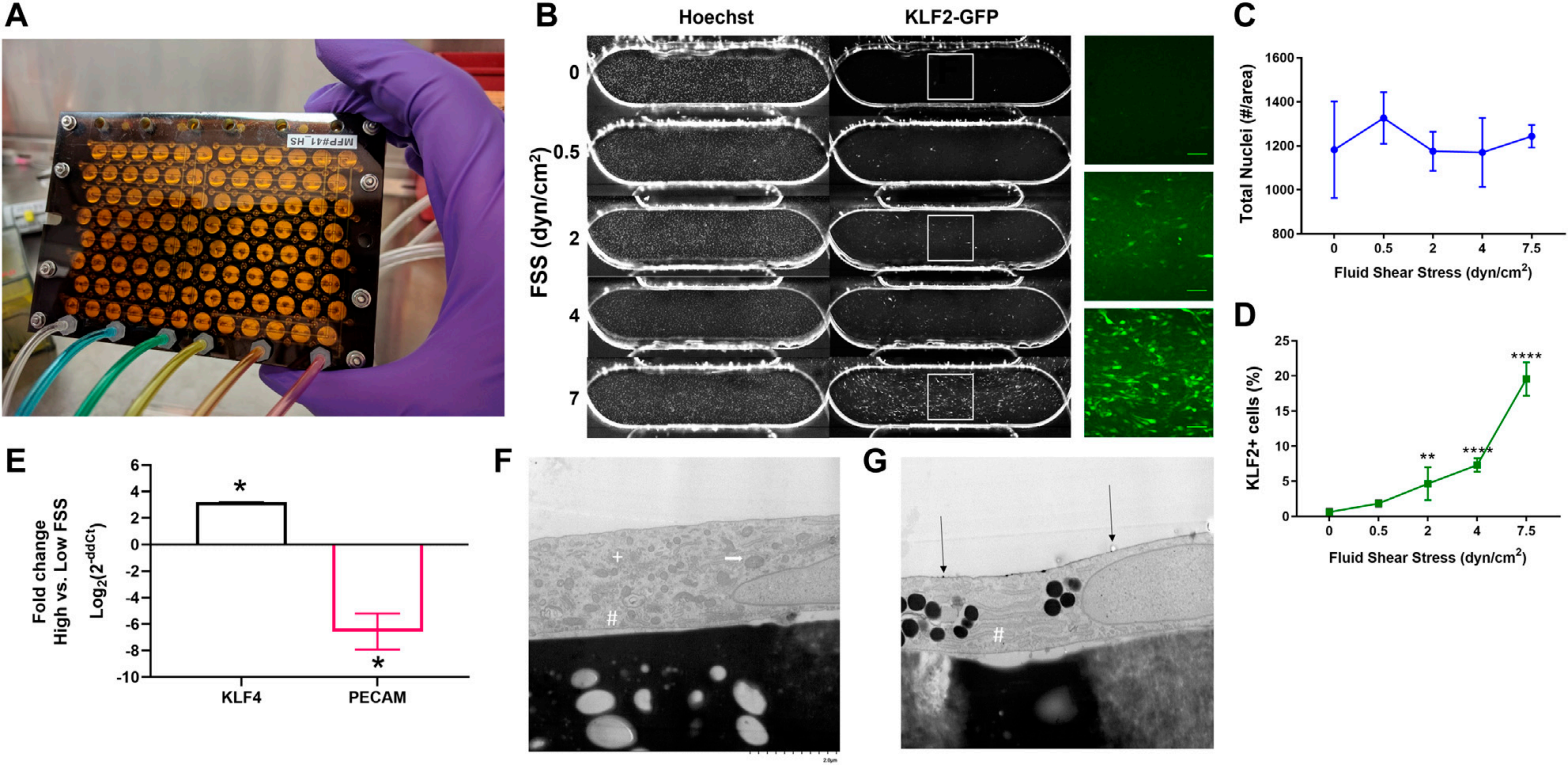
Validation of high flow pump and EC response to FSS. **(A)** Photo of the pneumatically actuated PREDICT96 high flow pump lid showing 96 arrayed micro-pumps. **(B)** Representative images of Hoechst stain and KLF2-GFP expression in reporter EC subjected to different FSS conditions. Insets show close-up of KLF2 for select conditions. Quantification of **(C)** total nuclei and **(D)** KLF2 expression as a function of FSS. **p < 0.005, ****p < 0.0001 for one-way ANOVA with Tukey’s *post hoc* test for *n* = 4-8 devices per condition. **(E)** Comparative gene expression for shear-responsive genes in retinal microvascular ECs; positive values indicate upregulation with exposure to high FSS (7 dyn/cm^2^) relative to low FSS (0.5 dyn/cm^2^), while negative fold change indicates downregulation. **(F)** Electron micrograph showing ultrastructure of retinal microvascular EC under low FSS, exemplified with cytoplasmic organelles, including rough endoplasmic reticulum (RER; #), Golgi (+), and mitochondria (white arrow). **(G)** Electron micrograph of EC under physiological FSS with more abundant RER and electron dense bodies consistent with lipids are observed. Gold particle labeling indicated by black arrows. Images at x7000 magnification.

Fluid shear stress (FSS) is a critical biomechanical cue for EC health and disease (Baeyens et al., 2016; Gimbrone and Garcia-Cardena, 2016). Prior to initiating the co-culture studies, we validated the ability of the high flow pump to modulate human EC response to fluid flow. The high flow pump (Figure 3A) had an average stroke volume of 11.7 µL (CV = 4.8%) and provided recirculating flow in one channel of each of the 96 dual-channel PREDICT96 devices. Independent control on the left and right halves of the pump allowed the study of two FSS regimes within a single experiment. In previous work, we have demonstrated alignment of EC with high FSS (Azizgolshani et al., 2021) and high viability of the co-culture model up to 2 weeks (Rogers et al., 2021). Here, we first validated the system using a reporter EC line which had been engineered to express fluorescently tagged KLF2, a well-known shear-responsive transcription factor (Slegtenhorst et al., 2018). Reporter EC were cultured under varying flow rates resulting in 0, 0.5, 2, 4, and 7.5 dyn/cm^2^ FSS for 48 h and then immediately imaged. KLF2 expression increased in a “dose-dependent” manner with increasing FSS (Figures 3B–D). We then analyzed the shear-responsive genes KLF4 and PECAM1 by RT-qPCR in the primary retinal microvascular EC to be used in co-culture studies after exposure to 0.5 dyn/cm^2^ and 7 dyn/cm^2^. KLF4 was significantly upregulated while PECAM1 was significantly downregulated in 7 dyn/cm^2^ compared to 0.5 dyn/cm^2^ conditions (Figure 3E).

Ultrastructural evaluation of cultured primary retinal microvascular EC in the PREDICT96 platform was successfully conducted using gold-particle labeling and electron microscopy. These experiments provided initial insights into the morphologic features of ECs that would be expected under different physiological conditions. EC under low FSS had a mixture of organelles including mitochondria, rough endoplasmic reticulum (RER), and Golgi (Figure 3F). When high FSS conditions were applied, the relative proportion of RER increased in relation to the other organelles such as the mitochondria and Golgi, and electron-dense bodies consistent with lipid were more prominent (Figure 3G). These observations are consistent with reorientation of the cells (Davies, 2009) and increased production of proteins such as VE-cadherin (Noria et al., 1999) associated with FSS.

Overall, these findings showed that the PREDICT96 system was capable of inducing a physiologically relevant range of FSS for our vascular model, as indicated by the differential responses of EC to low vs high FSS. We then defined 0.5 dyn/cm^2^ as “low” FSS and 7 dyn/cm^2^ as “high” FSS conditions for the remaining experiments. All remaining studies were conducted with the retinal microvascular EC and/or hDF.

### Altered activation response of perivascular cells in co-culture

**FIGURE 4:**
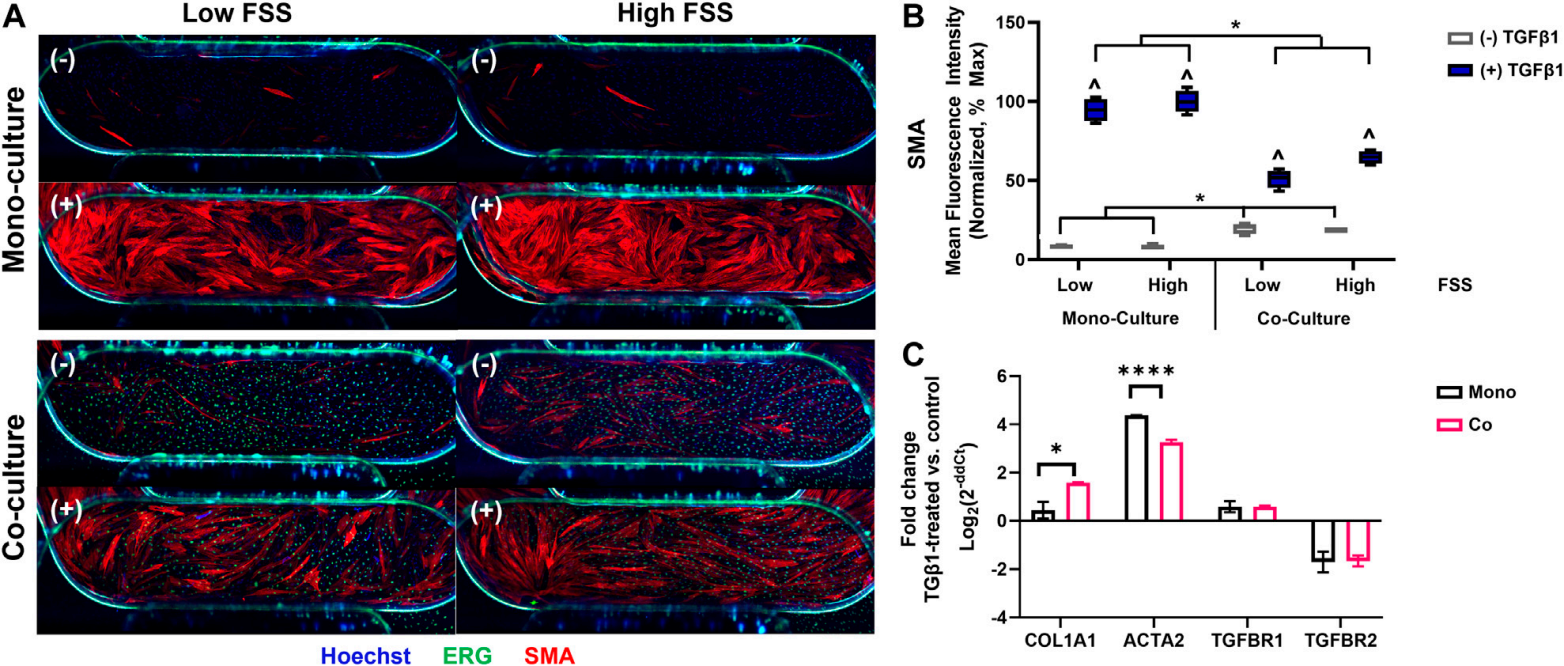
The response of fibroblasts to TGFB1 stimulation in co-culture with EC. **(A)** Representative immunofluorescence images of fibroblast mono- and co-culture conditions showing SMA (activated fibroblasts, red), ERG (EC nuclei, green), and Hoechst stain (all nuclei, blue). The images show primarily the device channel overlap region (4 mm × 1 mm), but fibroblasts can also be observed in the arms of the top channel (top corners of images) and EC nuclei in the arms of the bottom channel (bottom corners of images). (−) no TGFβ1 (+) 10 ng/mL TGFβ1. **(B)** Quantification of SMA mean fluorescence intensity (MFI) normalized to fibroblast mono-culture high FSS controls, which had the highest signal and assumed 100% activation. ^ *p* < 0.05 for TGFβ1-treated condition with respect to its untreated control, *p < 0.05 shown for additional comparisons of interest, two-way ANOVA with Sidak’s multiple comparisons test, *n* = 4 per condition. **(C)** Comparative gene expression for fibroblasts in mono-culture (black bars) and in co-culture with EC (red bars). Positive fold-change values indicate upregulation in TGFβ1-treated conditions with respect to controls; negative values indicate downregulation.

After selecting the perivascular cells and FSS conditions for further study, we then characterized the response of the EC-hDF co-culture model to TGFβ1 stimulation. A representative experiment is depicted in Figure 4 and representative images of cell nuclei are provided in Supplementary Figure S1. hDF, which were not directly exposed to FSS, did not exhibit any obvious orientation response. EC under low FSS had disorganized nuclei while under high FSS, nuclei became more uniform and oriented with the direction of flow. We next noted several interesting observations in the co-culture model compared to the hDF mono-cultures. First, there was often basal activation in the co-culture control conditions, in which no exogenous TGFβ1 had been added. However, despite basal effects, exogenous TGFβ1-stimulated activation of the hDF in co-culture with EC was significantly blunted compared to hDF mono-cultures. This observation was measured across multiple perivascular cell sources and independent experiments (data not shown). Furthermore, the effect was not altered by low vs high FSS (Figure 4B), suggesting that the presence of EC was the dominant factor in the response. In addition to quantification of SMA staining, we further corroborated the effect of co-culture by RT-qPCR (Figure 4C). Analysis of hDF gene expression in mono-culture and in co-culture with EC revealed that the *col1a1* gene was significantly upregulated in co-culture compared to mono-culture while the *acta2* gene (encoding SMA protein) was significantly downregulated. Of additional note, the genes for TGFβ receptors 1 and 2 were similar in mono- and co-cultured hDF, suggesting that reduced activation in co-culture was not due to alterations in receptor expression.

### Endothelial-derived secreted factors reduce activation of perivascular cell mono-cultures

**FIGURE 5:**
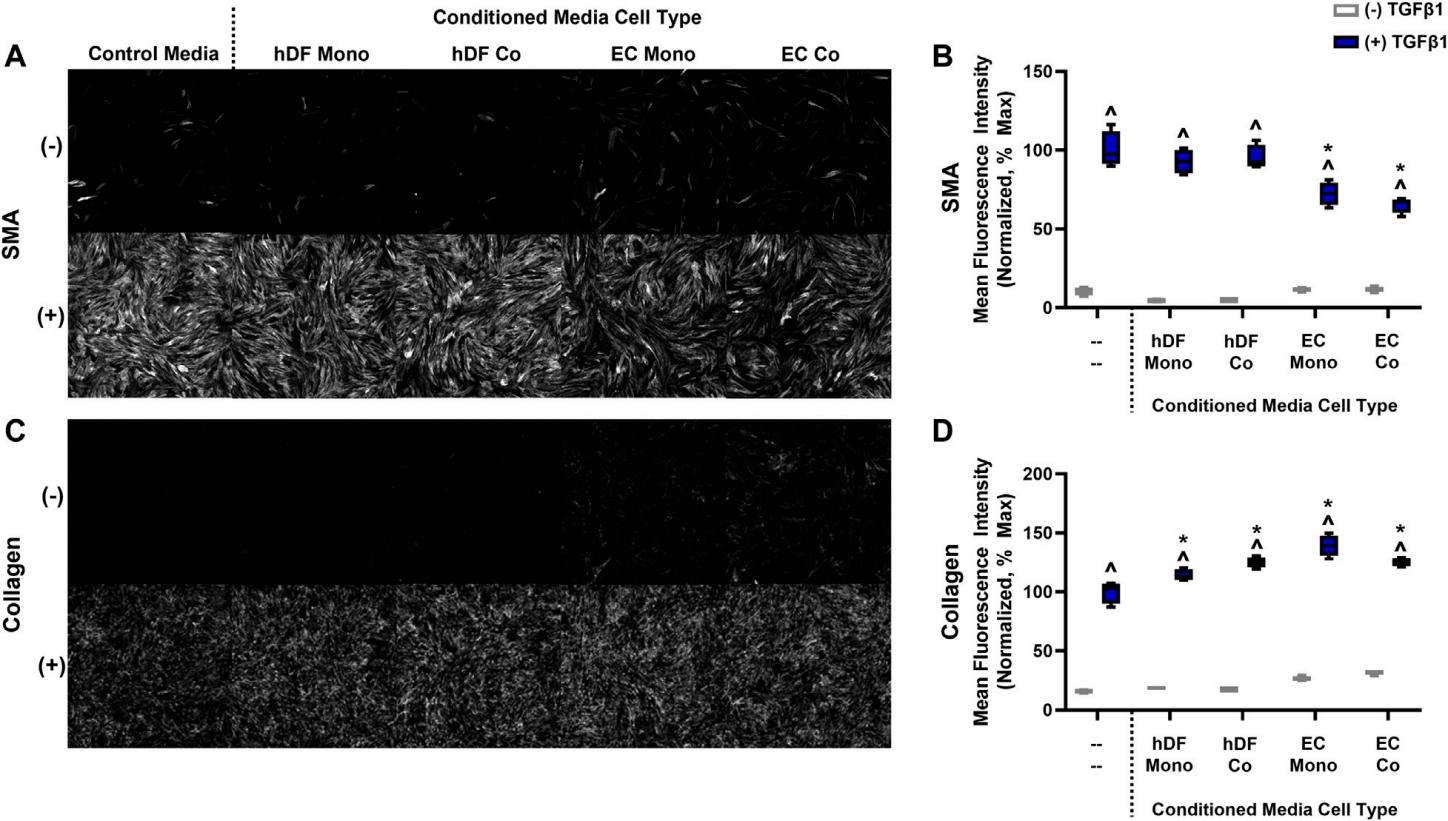
The effects of conditioned media on fibroblast activation in mono-culture. **(A)** Representative images of SMA staining for control media and conditioned media from hDF and EC, with or without 10 ng/mL TGFβ1. **(B)** Quantification of mean fluorescence intensity for SMA. **(C)** Representative images of fluorescent collagen probe for control media and conditioned media from hDF and EC, with or without 10 ng/mL TGFβ1. **(D)** Quantification of mean fluorescence intensity for collagen. For both graphs in B and D, data are normalized to the hDF in control media treated with TGFβ1, assumed 100% activation. ^ *p* < 0.05 for TGFβ1-treated conditions with respect to its untreated control, *p < 0.05 compared to the control media with TGFβ1, two-way ANOVA with Tukey’s multiple comparisons test, *n* = 4 per condition.

Intercellular cross-talk can occur via direct contacts or signaling via secreted factors (Méndez-Barbero et al., 2021). We examined whether soluble factors derived from EC may cause basal activation without TGFβ1 and/or blunted activation in the presence of TGFβ1. We collected conditioned media from EC mono-cultures, EC co-cultures, hDF mono-cultures, and hDF co-cultures exposed to high FSS and compared the activation responses of hDF mono-cultures treated with these various conditioned media (Figure 5). Interestingly, we found slight increased basal activation of hDF subjected to EC-derived conditioned media both in terms of SMA expression and collagen deposition compared to hDF conditioned media (Figures 5B, D gray), similar to our co-culture studies. In the presence of exogenous TGFβ1, SMA expression was significantly reduced in EC-derived conditioned media compared to hDF conditioned media and control (non-conditioned) media (Figures 5A, B blue). Collagen was slightly but significantly increased in all conditioned media compared to control media (Figures 5C, D blue).

We then sought to identify which EC-derived factors might affect the perivascular cell activation response. First, we considered that low level myofibroblast activation in control conditions could have been due to active TGFβ1 produced by the co-culture model, as described by previous studies (Sato et al., 1993). We found that EC produced significantly more TGFβ1 compared to hDF (Supplementary Figure S2) and at levels sufficient to induce activation, such as shown in Figure 2. The endogenous production of low levels of TGFβ1 by the EC likely explained the basal activation observed in the co-culture model in the absence of exogenous TGFβ1 stimulation.

**FIGURE 6:**
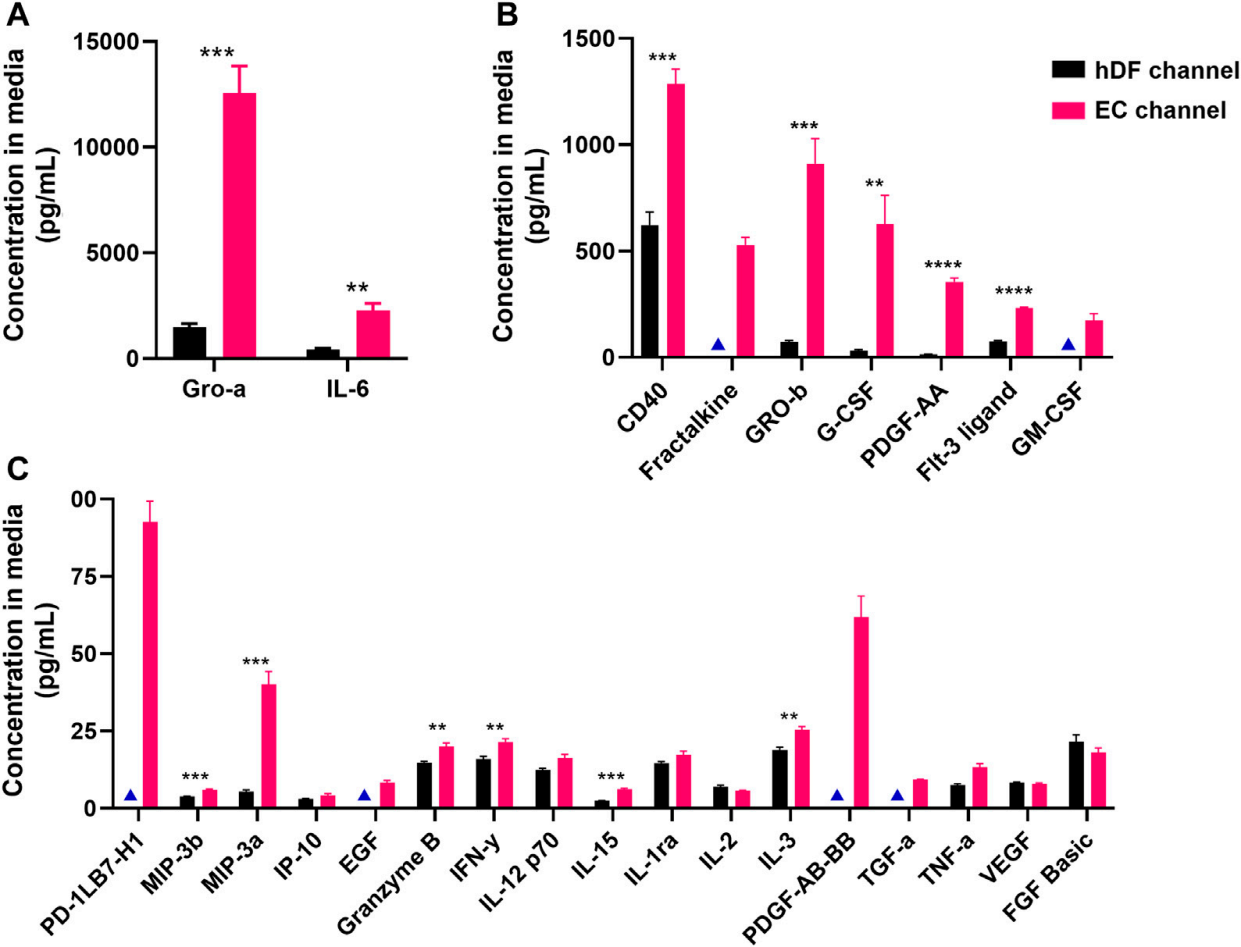
Secreted proteins detected in media from EC and hDF channels. A custom Luminex panel was used to detect secreted cytokines and growth factors. Media was harvested from the EC and hDF channels to determine cell type-enriched factors. Data is separated by **(A)** high, **(B)** medium, and **(C)** low concentration in the EC channel. Multiple unpaired t-tests were used to determine whether a factor was significantly enriched in the EC vs hDF-derived media. **p < 0.01, ***p < 0.001, ****p < 0.0001, N = 4 per condition. Blue triangles indicate conditions in which the analytes were below the limit of detection and not included in statistical analyses.

We then analyzed a 45-plex Luminex panel of secreted factors across our mono- and co-culture conditions to identify factors that may be unique to or enriched in the EC. Media was collected separately from the EC- and hDF-containing channels for analysis. Of the 45 factors analyzed, 26 produced detectable levels in the EC or hDF channel-derived media (Figure 6). Of these, 19 were significantly higher in the EC channel compared to the hDF channel: Gro-α, IL-6, CD40 Ligand, Fractalkine, Gro-β, G-CSF, PDGF-AA, Flt-3 Ligand, GM-CSF, PD-L1/B7-H1, MIP-3β, MIP-3α, EGF, Granzyme B, Interferon γ, IL-15, IL-3, PDGF-AB/BB, and TGFα. We ran experiments with hDF mono-cultures and recombinant human proteins to determine if the highly expressed factors from Figures 6A, B affected activation. There was no significant effect on SMA expression and only a modest, albeit significant, effect on collagen deposition in the presence of GRO-β, GRO-α, and Flt-3 Ligand (Supplementary Figure S3).

In addition, we identified differentially expressed genes in the EC vs hDF populations from our scRNA-seq data (Table 3). Note that 6 of the 9 highly expressed factors identified in the Luminex panel also had significant gene upregulation in EC vs hDF populations: CXCL2/GRO-β, CXCL1/GRO-α, CSF3/G-CSF, PDGFA, CD40, CX3CL1/Fractalkine. The TGFβ1 gene was also enriched in the EC population, in agreement with our ELISA study demonstrating increased TGFβ1 production by EC. However, a number of other factors and families were identified, such as members of the TNF super family, FGFs, and interleukins.

**TABLE 3:** Differentially expressed genes in EC–cytokines and growth factors.

Gene short name	Entrez gene ID	Number of EC expressing	log2 fold change
KDR	3791	1001	7.2
PDGFB	5155	666	6.4
JAG2	3714	1012	6.2
CSF2RB	1439	496	5.5
TNFSF15	9966	605	5.4
BMP6	654	518	5.2
LTB	4050	153	5.2
TNFSF4	7292	606	5.2
TNFRSF4	7293	249	4.7
TNFSF10	8743	506	4.6
IL3RA	3563	230	4.4
ACVRL1	94	4872	4.3
GDF3	9573	190	4.3
TNFRSF25	8718	1061	4.1
EFEMP1	2202	742	4.0
TNFRSF21	27242	2177	3.8
FLT1	2321	1,340	3.7
CCL2	6347	3234	3.6
BMP4	652	2747	3.5
CXCL8	3576	636	3.4
PDGFD	80310	1584	3.4
IL4R	3566	1469	3.3
PGF	5228	4276	3.3
HBEGF	1839	1,132	3.1
BMP2	650	517	3.0
TNFSF9	8744	510	3.0
CCN2	1490	4617	2.9
TNFRSF10A	8797	1188	2.8
KIT	3815	118	2.7
CXCL2	2920	636	2.6
NRG3	10718	52	2.6
TNFRSF10C	8794	693	2.6
TGFBR2	7048	5625	2.6
TNFRSF11A	8792	145	2.5
IL1A	3552	204	2.5
CXCL1	2919	2277	2.4
TNFSF18	8995	120	2.4
TNFRSF1B	7133	1,059	2.4
FLT4	2324	65	2.4
FGF12	2257	77	2.4
GDF7	151449	282	2.3
CSF3	1440	72	2.2
IL6ST	3572	7464	2.1
IL18R1	8809	127	2.0
GMFG	9535	230	2.0
JAG1	182	1712	1.9
TNFRSF10B	8795	4765	1.9
EPOR	2057	771	1.9
BMPR2	659	5647	1.8
PDGFA	5154	2593	1.8
CD40	958	876	1.8
AMH	268	161	1.8
TGFB2	7042	674	1.8
NRG1	3084	1,673	1.8
OSGIN2	734	2061	1.8
FGF18	8817	206	1.8
TNFRSF10D	8793	2247	1.7
MET	4233	1,642	1.7
IL6R	3570	418	1.7
TGFB1	7040	6463	1.7
GDF15	9518	4070	1.7
OSGIN1	29948	1,413	1.7
MDK	4192	8472	1.6
GRN	2896	8524	1.6
CXCL16	58191	555	1.6
PLEKHO2	80301	2411	1.6
CX3CL1	6376	16	1.6
BMPR1B	658	89	1.5
ACVR1B	91	806	1.5
IFNAR2	3455	3562	1.4
INHBA	3624	2181	1.4
PSPN	5623	117	1.4
TNFRSF14	8764	3355	1.3
IL13RA1	3597	4604	1.3
RELT	84957	626	1.3
RABEP2	79874	2912	1.3
NRG2	9542	74	1.2
ADA2	51816	579	1.1
HDGFL3	50810	3645	1.1
LIFR	3977	1,437	1.1
IFNGR2	3460	689	1.1
ADA2	51816	579	1.1
HDGFL3	50810	3645	1.1
CD320	51293	5948	1.0

### scRNA-seq reveals sub-populations of EC and hDFs, including acta2-enriched clusters

To examine the physiological status of cells within the model and to identify potential crosstalk between cell types, we employed scRNA-seq. While not as sensitive as bulk RNA sequencing, scRNA-seq is well-suited for identifying and quantifying cellular heterogeneity within tissues (Patel et al., 2014; Choi and Kim, 2019). The vascular models were constructed with co-cultured EC and hDF, as well as mono-cultures of EC and hDF. Some of the samples were treated with TGFβ1. We isolated cells from the devices at 5 days post-TGFβ1 stimulation and performed scRNA-seq on the mixed populations. First, we confirmed sufficient cell numbers and viability (Supplementary Figure S4). Extractions typically yielded 1,500–3,500 cells per channel, with >75% viability in most cases. We anticipated heterogeneous gene expression within the EC and hDF cell populations from the various conditions, reflecting their responses to changes in the local signaling environment induced by co-culture and/or TGFβ1 stimulation.

**FIGURE 7:**
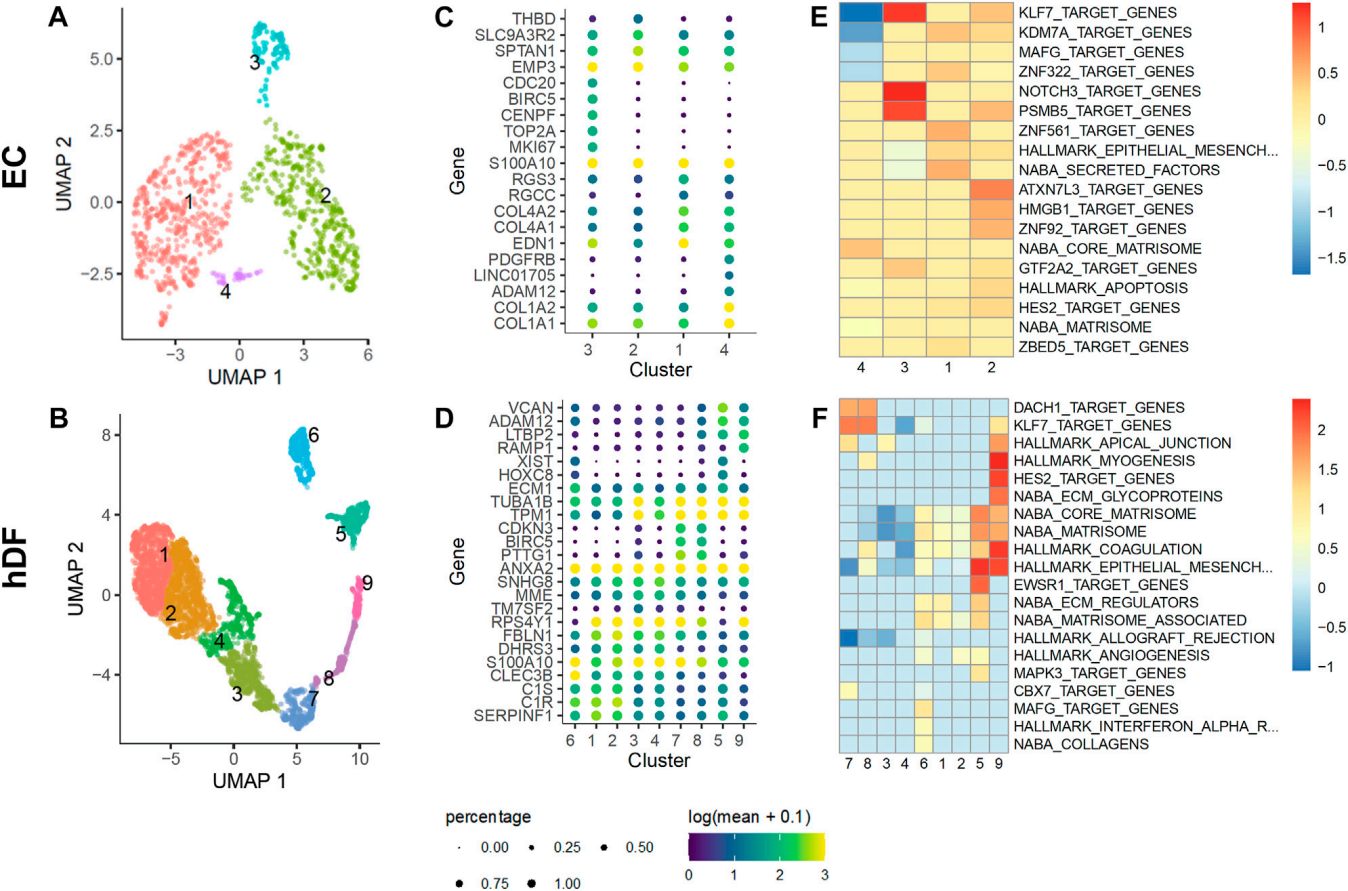
Identification of EC and hDF sub-populations by scRNA-seq. EC and hDF were isolated from co-culture models, encapsulated using a 10X Chromium flow cell, and sequenced. Cell types were identified from gene expression signatures by clustering on highly-variable genes (see methods). Distinct cell subpopulations for both **(A)** EC and **(B)** hDF were observed. Markers for the clusters within **(C)** EC and **(D)** hDF sub-populations highlight significant phenotypic heterogeneity, including proliferation, differentiation, and wound repair. Distinct gene programs co-expressed within each cluster for **(E)** EC and **(F)** hDF enrich MsigDB terms that suggest both unique and overlapping biological processes as cells within each subpopulation adapt and respond to their microenvironment.

Both cell types clustered into distinct cell sub-populations with different characteristics (Figure 7). The number of clusters correlated with the expected heterogeneity of the cell types. EC formed four clusters while hDF formed nine clusters (Figures 7A, B). Each cell type had at least one cluster expressing markers of proliferation (TOP2A, BIRC5, TK1, and/or CDC20) (Figures 7C, D). The non-proliferating populations of each cell type expressed signature genes consistent with responses to extracellular cues. Non-proliferating EC separated into two cell clusters. The EC subpopulation in cluster #1 expressed cytokines such as MDK, CCL2, and GDF15 which control cell growth and differentiation, and promote angiogenesis. The EC subpopulation in cluster #2 expressed genes such as CAV1, ADAM15, and ERK signaling targets such as VIM and CCDN3 which control cell proliferation, migration, and responses to stress. The observation of these two distinct EC subpopulations is reminiscent of angiogenesis and neovascularization, two EC-dependent processes controlled by many extracellular factors, including TGFβ1 and VEGF.

**FIGURE 8:**
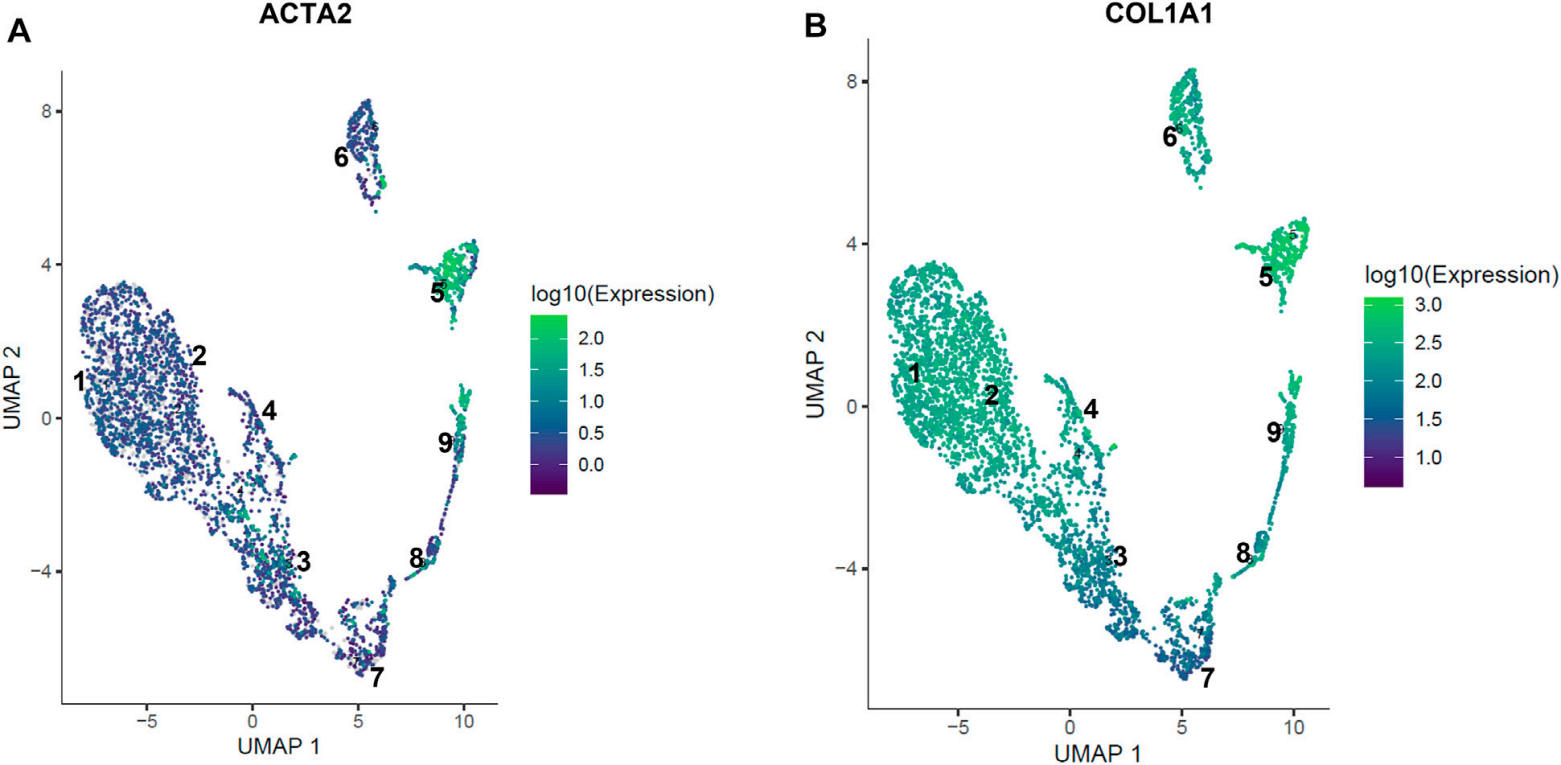
Expression of acta2 and col1a1 in hDF sub-populations. Localized expression of markers of fibroblast activation are observed in the hDF cell subpopulation by scRNA-seq. **(A)** The gene for alpha smooth muscle actin (ACTA2) expression is highly specific for clusters #5 and #9. **(B)** Collagen type 1 (COL1A1) is co-expressed with ACTA2 in clusters #5 and #9 consistent with an activated myofibroblast phenotype. Other COL1A1 expressing cells are not expressing ACTA2, suggesting differentiated matrix-producing fibroblasts that have not fully transitioned to a myofibroblast phenotype.

The hDF cell subpopulation had clusters expressing *acta2* and ECM-related genes, clusters #5 and #9, that were distinct from separate clusters of proliferating cells (Figure 8). In addition, there were clusters of cells that appeared largely quiescent (clusters #3 and #4). Non-proliferating and non-activated hDF formed a supercluster expressing high levels of DCN and collagen precursors. Interestingly, while one part of the supercluster, cluster #4, was strongly quiescent, marked by downregulation of cell proliferation and cytoskeletal rearrangement genes, neighboring clusters #1 and #2 expressed genes related to complement activation (C1R, C1S, CLEC3B). This latter set of cells also expressed higher levels of ECM-associated genes, such as ITGB1, MMP2, and SERPINE2, suggesting a possible link between activation of innate immunity and onset of fibrosis.

## Discussion

The activation of perivascular cells to a myofibroblast phenotype is an early event in fibrosis that is common across organ types (Greenhalgh et al., 2013). While perivascular cells have been of particular interest as targets of novel therapeutics, other cell types that interact with pro-fibrotic perivascular cells should be considered, particularly those that could limit a pathogenic response. Studies of liver fibrosis have revealed the importance of liver sinusoidal EC in maintaining stellate cell quiescence (DeLeve et al., 2008; Poisson et al., 2017). The goal of this work was to determine whether this phenomenon was generalizable (i.e., occurring in other types of EC-perivascular cell interactions) and to gain insight into potential mechanisms. The key findings of our study are as follows: 1) We found that co-culture of hDF with microvascular EC reduced TGFβ1-induced myofibroblast activation. Specifically, SMA expression was reduced at the protein and gene expression levels as determined by immunofluorescence staining and RT-qPCR, respectively. 2) The effect of EC was at least partially due to secreted factors as determined by conditioned media studies, although the specific factor(s) remain elusive. 3) Single cell RNA-seq studies revealed a number of differentially expressed factors in EC vs hDF that could be explored in future work. In addition, scRNA-seq identified subpopulations of EC and hDF, most notably acta2-and collagen-expressing hDF clusters indicative of the myofibroblast activated state as well as quiescent hDF subpopulations.

The vascular model and experimental workflow was designed to achieve some level of activation in the both the hDF mono-culture and EC-hDF co-cultures. This goal required optimization of the co-culture media, order of cell seeding, and timing of TGFβ1 stimulation. In our initial studies, we explored various sources of perivascular cells: lung fibroblasts, dermal fibroblasts, and retinal pericytes. Interestingly, not all of these cell sources had the same response to TGFβ1, with lung fibroblasts being “high” responders, hDF being “mid” responders, and RPC being “low” responders (Figure 2). These differential responses could be due to variations across donors, ages, and/or tissue sources. We were also able to rule out potential EC activation (endothelial to mesenchymal transition) as we saw no TGFβ1-induced expression of SMA in the EC source selected for this study. Interestingly, we also found that the model was difficult to perturb if TGFβ1 was added more than 24–30 h after seeding the perivascular cells. Therefore, in early experiments we selected the approach of seeding the EC first so that the perivascular cell source could be treated with TGFβ1 within an optimal time frame. We also explored methods to enhance myofibroblast activation in co-culture by attempting to simultaneously induce EC injury with TNFα or thrombin. However, these approaches did not affect hDF activation. Additionally, we found that FSS was required for reproducible outcomes in the co-culture model, as statically cultured models tended to have high basal activation and/or inconsistent responses to exogenous TGFβ1 (data not shown).

The PREDICT96 high flow pump is unique in that it can apply physiologically relevant FSS to 96 independent microfluidic vascular tissues in the PREDICT96 plate. We confirmed that EC were responsive to FSS using a KLF2 reporter cell line, morphological changes (qualitative elongation and alignment with the direction of fluid flow), ultrastructural changes, and the expression of two shear-responsive genes (Figure 3). KLF2 and KLF4 are known to increase with physiological FSS (Hamik et al., 2007; Fan et al., 2017). Others have reported that low FSS can induce an inflammatory or atheroprone state in ECs, while high FSS promotes a healthy phenotype (Dai et al., 2004; Ruze et al., 2018; Xie et al., 2020). Accordingly, PECAM1 (CD31), an inflammatory and shear-responsive molecule in EC (Woodfin et al., 2007) was downregulated with high FSS compared to low FSS. Of note, we found that FSS, at least at the two flow rates studied, did not appear to be a major factor in the hDF activation response: activation was similarly blunted in low and high FSS co-culture models compared to hDF mono-cultures (Figure 4). This result suggested that the presence of EC was the most significant variable affecting the hDF activation response. However, given the considerable range of physiological FSS in the human vascular system (Hathcock, 2006), it is possible that different levels of FSS could have a significant effect and would be interesting to study in future work. Furthermore, we acknowledge that other potentially relevant variables may change with flow, such as mixing, oxygenation, or transport of nutrients, which could also affect cell response.

The primary discovery of this work was that TGFβ1-induced activation of hDF in co-culture with EC was consistently blunted compared to the hDF mono-culture response. We measured variability in our platform for key conditions within and across plates (Supplementary Table S1) and generally found it to be low or within acceptable ranges for biological systems (4%–18%), with the highest variability observed in hDF mono-cultures without TGFβ1. Considering the methods of cell-cell communication, the potential mechanisms could be via secreted factors (paracrine or autocrine) or via direct cell-cell contacts. Due to limitations of electron microscopy, we were unable to observe whether the two cell types could make direct contact through the microporous membrane. However, in preliminary studies of direct contact co-culture with both cell types in well plates, hDF had significant and robust activation without the addition of exogenous TGFβ1 (data not shown). Others have found that EC-smooth muscle cell co-cultures produce active TGFβ1 (Sato et al., 1993) which may explain this phenomenon in our own direct co-culture studies, as well as the low basal activation observed in the “indirect” co-culture configuration in PREDICT96 devices. Therefore, we cannot rule out direct contact through the microporous membrane as a potential, but likely minor, mechanism of EC-hDF communication in our model.

Our conditioned media studies revealed that EC-derived media, regardless of mono- or co-culture, consistently blunted hDF mono-culture activation to similar levels observed in the co-culture model (Figures 4, 5). In contrast, hDF-conditioned media, regardless of mono- or co-culture, had no effect on hDF activation in mono-culture. Taken together, this data suggested that the blunted activation effect was due to paracrine signaling from the EC and did not appear to involve autocrine signaling from the hDF. We note that the studies of mono-cultured fibroblast response to conditioned media were carried out in 96 well plates for simplicity. Although preliminary mono-culture experiments did not indicate a difference in activation behavior in 96 well plates vs PREDICT96, we have not completed detailed comparisons of these culture formats, but believe it could be valuable in future work. In addition, we did not include fibroblast-fibroblast co-cultures, which could also provide additional insights in future studies.

We then identified potential EC-derived factors by Luminex and scRNA-seq. The Luminex panel identified 19 factors that were significantly enriched in EC channel-derived media compared to hDF-derived media. Of these, two factors (GROα, IL-6) were highly expressed at ∼2,280–12,500 pg/mL, seven factors (CD40, Fractalkine, GROβ, G-CSF, PDGF-AA, Flt-3 Ligand, GM-CSF) were mid-range at 173–1,285 pg/mL, and the remaining 10 factors were low abundance at < 100 pg/mL. Interestingly, some of these growth factors and cytokines have been associated with fibrosis, such as PDGF (Cesta et al., 2010; Borkham-Kamphorst and Weiskirchen, 2016; Wang et al., 2017), GROα/CXCL1, and GROβ/CXCL2 (Wynn, 2011; Wu et al., 2021), although we note that many of these studies have been carried out in animal models. Other factors, such as G-CSF and GM-CSF, are associated with inhibition of fibrosis (Moore et al., 2000; Zhao et al., 2020). However, we found that none of the 9 high to mid-range EC-derived factors significantly affected SMA expression in hDF mono-cultures stimulated with TGFβ1, although GROβ, GROα, and Flt-3 Ligand had modest effects on deposited collagen. It is possible that lower abundance factors such as TNFα and FGF-2, which are known to reduce myofibroblast activation (Arancibia et al., 2013; Dolivo et al., 2017), could be playing a role and potentially in concert with other low abundance factors. It is also possible that EC-secreted factors not identified by the Luminex panel (additional growth factors or cytokines, soluble receptors (Smith et al., 1999), additional isoforms of TGFβ or members of the TGFβ superfamily, microRNAs, lipids, ECM-derived or sequestered factors) are responsible for the effects on hDF, but a more comprehensive experimental study was beyond the scope of this work.

The scRNA-seq data offers an opportunity to generate additional hypotheses where expression of SMA by hDF could be modulated by cross-talk between EC and hDF. By looking at differential gene expression between EC and hDF, we identified a set of EC-specific genes expressed in co-culture. We then subset this list of EC-specific genes to those expected to be involved in cellular cross-talk mechanisms: cytokines, growth factors, and ECM proteins (Table 3). While some of the genes corroborated the Luminex data, two compelling alternative hypotheses also emerged. Heparin binding EGF-like growth factor (HBEGF) is moderately expressed by EC and is known to induce proliferation in fibroblasts, which could inhibit SMA expression (Kirkland et al., 1998). In addition, JAG2 is highly expressed by EC and regulates cell fate decisions via Notch signaling. JAG1 and JAG2 have been shown to have distinct biological activities in rapidly proliferating cells (Choi et al., 2009), and the NOTCH3 receptor is selectively expressed by hDF in our model (Supplementary Figure S5). NOTCH3 has been shown to control fibroblast differentiation and mediate inflammation (Wei et al., 2020). While neither of these hypotheses can definitively rule out more complex mechanisms, they exemplify some of the ways that a co-culture model such as ours can be used to investigate mechanisms of cellular cross-talk and their influences on complex disease states, especially at early stages of pathogenesis.

In addition to the limitations described above, we note the following aspects of our model which could be explored in future work. The cells used were not donor-, age-, or sex-matched due to what was commercially available at the time the work was carried out. Although some of our preliminary work with the microvascular EC and RPC were tissue source-matched (retinal), most of our studies used the “mid-responding” hDF and thus were not tissue-matched. It is well-known that organ-specific EC have unique phenotypes that may influence cellular and tissue responses (Aird, 2012; Jambusaria et al., 2020; Gunawardana et al., 2021), which would be interesting to study in the future. In particular, expanding the platform to include tissue-matched EC-perivascular cell co-cultures would provide mechanistic insights into either organ-agnostic or tissue-specific responses to pro-fibrotic perturbations. Regarding fibroblasts, these cells are a diverse population within and across organs (Lendahl et al., 2022), and can take on many states or phenotypes. Our studies focused on the evaluation of activation via SMA and collagen, although we acknowledge that the impact of EC on fibroblast behavior could very well extend beyond these measurements. Most of our experiments used SMA expression as measure of activation, which did not always correlate with collagen deposition. Given that these studies were conducted over a relatively short timeframe post-TGFβ1 exposure (72–96 h), longer-term effects on ECM deposition should be measured to further characterize myofibroblast state, as well as other indicators such as gene expression profiles or contractility. In addition, our model did not include immune cells, and we note that macrophages would be an important next step for the model, given their role in fibrosis (Wynn and Barron, 2010). We believe that earlier time points or a time course study utilizing scRNA-seq analyses would also provide further valuable insight into the influence of EC and cellular cross-talk in myofibroblast activation.

In summary, we have developed a human vascular organ-on-chip co-culture model with physiological FSS that has provided insight into the role of EC in modulating the early TGFβ1-induced activation responses of perivascular cells. The throughput nature of the PREDICT96 platform could enable exploration of age and sex differences, and organ-specific pairings of microvascular EC and pericytes/fibroblasts (heart, lung, skin, liver, etc.) in future work. Given the influence of EC on myofibroblast activation, EC-derived factors warrant further investigation for anti-fibrotic therapies.

## Data Availability

The single cell RNA-sequencing data presented in this study are deposited in the Single Cell Portal, accession number SCP2301. All other data are contained within the main article and Supplementary Material. Further inquiries can be directed to the corresponding author.

## References

[B1] Abdalla M. Goc A. Segar L. Somanath P. R. (2013). Akt1 mediates α-smooth muscle actin expression and myofibroblast differentiation via myocardin and serum response factor. J. Biol. Chem. 288, 33483–33493. 10.1074/jbc.m113.504290 24106278 PMC3829193

[B2] Aird W. C. (2012). Endothelial cell heterogeneity. Csh Perspect. Med. 2, a006429. 10.1101/cshperspect.a006429 PMC3253027 22315715

[B3] Aper S. J. A. Spreeuwel A. C. C. V. Turnhout M. C. V. Linden A. J. V. Pieters P. A. Zon N. L. L. V. (2014). Colorful protein-based fluorescent probes for collagen imaging. Plos One 9, e114983. 10.1371/journal.pone.0114983 25490719 PMC4260915

[B4] Arancibia R. Oyarzún A. Silva D. Tobar N. Martínez J. Smith P. C. (2013). Tumor necrosis factor-α inhibits transforming growth factor-β-stimulated myofibroblastic differentiation and extracellular matrix production in human gingival fibroblasts. J. Periodontol. 84, 683–693. 10.1902/jop.2012.120225 22813343

[B5] Azizgolshani H. Coppeta J. R. Vedula E. M. Marr E. E. Cain B. P. Luu R. J. (2021). High-throughput organ-on-chip platform with integrated programmable fluid flow and real-time sensing for complex tissue models in drug development workflows. Lab. Chip 21, 1454–1474. 10.1039/d1lc00067e 33881130

[B6] Baeyens N. Bandyopadhyay C. Coon B. G. Yun S. Schwartz M. A. (2016). Endothelial fluid shear stress sensing in vascular health and disease. J. Clin. Invest. 126, 821–828. 10.1172/jci83083 26928035 PMC4767335

[B7] Bainbridge P. (2013). Wound healing and the role of fibroblasts. J. Wound Care 22, 407–408. 10.12968/jowc.2013.22.8.407 23924840

[B8] Borkham-Kamphorst E. Weiskirchen R. (2016). The PDGF system and its antagonists in liver fibrosis. Cytokine Growth F. R. 28, 53–61. 10.1016/j.cytogfr.2015.10.002 26547628

[B9] Braddock M. Schwachtgen J. L. Houston P. Dickson M. C. Lee M. J. Campbell C. J. (1998). Fluid shear stress modulation of gene expression in endothelial cells. Physiology 13, 241–246. 10.1152/physiologyonline.1998.13.5.241 11390796

[B10] Buchanan C. F. Verbridge S. S. Vlachos P. P. Rylander M. N. (2014). Flow shear stress regulates endothelial barrier function and expression of angiogenic factors in a 3D microfluidic tumor vascular model. Cell. Adhes. Migr. 8, 517–524. 10.4161/19336918.2014.970001 PMC4594487 25482628

[B11] Cao J. Spielmann M. Qiu X. Huang X. Ibrahim D. M. Hill A. J. (2019). The single-cell transcriptional landscape of mammalian organogenesis. Nature 566, 496–502. 10.1038/s41586-019-0969-x 30787437 PMC6434952

[B12] Carter J. K. Friedman S. L. (2022). Hepatic stellate cell-immune interactions in NASH. Front. Endocrinol. 13, 867940. 10.3389/fendo.2022.867940 PMC9218059 35757404

[B13] Cesta M. F. Ryman-Rasmussen J. P. Wallace D. G. Masinde T. Hurlburt G. Taylor A. J. (2010). Bacterial lipopolysaccharide enhances PDGF signaling and pulmonary fibrosis in rats exposed to carbon nanotubes. Am. J. Resp. Cell. Mol. 43, 142–151. 10.1165/rcmb.2009-0113oc PMC2937228 19738159

[B14] Choi K. Ahn Y. H. Gibbons D. L. Tran H. T. Creighton C. J. Girard L. (2009). Distinct biological roles for the Notch ligands jagged-1 and jagged-2. J. Biol. Chem. 284, 17766–17774. 10.1074/jbc.m109.003111 19398556 PMC2719415

[B15] Choi Y. H. Kim J. K. (2019). Dissecting cellular heterogeneity using single-cell RNA sequencing. Mol. Cells 42, 189–199. 10.14348/molcells.2019.2446 30764602 PMC6449718

[B16] Chung J. Kim K. H. Yu N. An S. H. Lee S. Kwon K. (2022). Fluid shear stress regulates the landscape of microRNAs in endothelial cell-derived small extracellular vesicles and modulates the function of endothelial cells. Int. J. Mol. Sci. 23, 1314. 10.3390/ijms23031314 35163238 PMC8836123

[B17] Colliva A. Braga L. Giacca M. Zacchigna S. (2020). Endothelial cell–cardiomyocyte crosstalk in heart development and disease. J. Physiol. 598, 2923–2939. 10.1113/jp276758 30816576 PMC7496632

[B18] Dai G. Kaazempur-Mofrad M. R. Natarajan S. Zhang Y. Vaughn S. Blackman B. R. (2004). Distinct endothelial phenotypes evoked by arterial waveforms derived from atherosclerosis-susceptible and -resistant regions of human vasculature. Proc. Natl. Acad. Sci. 101, 14871–14876. 10.1073/pnas.0406073101 15466704 PMC522013

[B19] Dardik A. Yamashita A. Aziz F. Asada H. Sumpio B. E. (2005). Shear stress-stimulated endothelial cells induce smooth muscle cell chemotaxis via platelet-derived growth factor-BB and interleukin-1alpha. J. Vasc. Surg. 41, 321–331. 10.1016/j.jvs.2004.11.016 15768016

[B20] Davies P. F. (2009). Hemodynamic shear stress and the endothelium in cardiovascular pathophysiology. Nat. Clin. Pract. Card. 6, 16–26. 10.1038/ncpcardio1397 PMC2851404 19029993

[B21] DeLeve L. D. Wang X. Guo Y. (2008). Sinusoidal endothelial cells prevent rat stellate cell activation and promote reversion to quiescence. Hepatology 48, 920–930. 10.1002/hep.22351 18613151 PMC2695448

[B22] Dewidar B. Meyer C. Dooley S. Meindl-Beinker N. (2019). TGF-Β in hepatic stellate cell activation and liver fibrogenesis—updated 2019. Cells 8, 1419. 10.3390/cells8111419 31718044 PMC6912224

[B23] Dolivo D. M. Larson S. A. Dominko T. (2017). FGF2-mediated attenuation of myofibroblast activation is modulated by distinct MAPK signaling pathways in human dermal fibroblasts. J. Dermatol Sci. 88, 339–348. 10.1016/j.jdermsci.2017.08.013 28899582 PMC5701866

[B24] Duffield J. S. (2014). Cellular and molecular mechanisms in kidney fibrosis. J. Clin. Invest. 124, 2299–2306. 10.1172/jci72267 24892703 PMC4038570

[B25] Fan Y. Lu H. Liang W. Hu W. Zhang J. Chen Y. E. (2017). Krüppel-like factors and vascular wall homeostasis. J. Mol. Cell. Biol. 9, 352–363. 10.1093/jmcb/mjx037 28992202 PMC5907833

[B26] Feaver R. E. Cole B. K. Lawson M. J. Hoang S. A. Marukian S. Blackman B. R. (2016). Development of an *in vitro* human liver system for interrogating nonalcoholic steatohepatitis. Jci Insight 1, e90954. 10.1172/jci.insight.90954 27942596 PMC5135271

[B27] Fleming I. Fisslthaler B. Dixit M. Busse R. (2005). Role of PECAM-1 in the shear-stress-induced activation of Akt and the endothelial nitric oxide synthase (eNOS) in endothelial cells. J. Cell. Sci. 118, 4103–4111. 10.1242/jcs.02541 16118242

[B28] Frangogiannis N. G. (2020). Cardiac fibrosis. Cardiovasc Res. 117, 1450–1488. 10.1093/cvr/cvaa324 PMC8152700 33135058

[B29] Galbusera M. Zoja C. Donadelli R. Paris S. Morigi M. Benigni A. (1997). Fluid shear stress modulates von Willebrand factor release from human vascular endothelium. Blood 90, 1558–1564. 10.1182/blood.v90.4.1558 9269774

[B30] Gimbrone M. A. Garcia-Cardena G. (2016). Endothelial cell dysfunction and the pathobiology of atherosclerosis. Circ. Res. 118 (4), 620–636. 10.1161/CIRCRESAHA.115.306301 26892962 PMC4762052

[B31] Greenhalgh S. N. Iredale J. P. Henderson N. C. (2013). Origins of fibrosis: Pericytes take centre stage. F1000prime Rep. 5, 37. 10.12703/p5-37 24049641 PMC3768328

[B32] Gunawardana H. Romero T. Yao N. Heidt S. Mulder A. Elashoff D. A. (2021). Tissue-specific endothelial cell heterogeneity contributes to unequal inflammatory responses. Sci. Rep-uk 11, 1949. 10.1038/s41598-020-80102-w PMC7820348 33479269

[B33] Hamik A. Lin Z. Kumar A. Balcells M. Sinha S. Katz J. (2007). Kruppel-like factor 4 regulates endothelial inflammation. J. Biol. Chem. 282, 13769–13779. 10.1074/jbc.m700078200 17339326

[B34] Hathcock J. J. (2006). Flow effects on coagulation and thrombosis. Arterioscler. Thromb. Vasc. Biol. 26, 1729–1737. 10.1161/01.atv.0000229658.76797.30 16741150

[B35] Henderson N. C. Rieder F. Wynn T. A. (2020). Fibrosis: From mechanisms to medicines. Nature 587, 555–566. 10.1038/s41586-020-2938-9 33239795 PMC8034822

[B36] Hernandez-Gea V. Friedman S. L. (2011). Pathogenesis of liver fibrosis. Pathol. Mech. Dis. 6, 425–456. 10.1146/annurev-pathol-011110-130246 21073339

[B37] Herrera J. Henke C. A. Bitterman P. B. (2018). Extracellular matrix as a driver of progressive fibrosis. J. Clin. Invest. 128, 45–53. 10.1172/jci93557 29293088 PMC5749528

[B38] Hinz B. (2016). Myofibroblasts. Exp. Eye Res. 142, 56–70. 10.1016/j.exer.2015.07.009 26192991

[B39] Huang E. Peng N. Xiao F. Hu D. Wang X. Lu L. (2020). The roles of immune cells in the pathogenesis of fibrosis. Int. J. Mol. Sci. 21, 5203. 10.3390/ijms21155203 32708044 PMC7432671

[B40] Hung C. Linn G. Chow Y. H. Kobayashi A. Mittelsteadt K. Altemeier W. A. (2013). Role of lung pericytes and resident fibroblasts in the pathogenesis of pulmonary fibrosis. Am. J. Resp. Crit. Care 188, 820–830. 10.1164/rccm.201212-2297oc PMC3826269 23924232

[B41] Jambusaria A. Hong Z. Zhang L. Srivastava S. Jana A. Toth P. T. (2020). Endothelial heterogeneity across distinct vascular beds during homeostasis and inflammation. Elife 9, 51413. 10.7554/elife.51413 PMC7002042 31944177

[B42] Kadohama T. Nishimura K. Hoshino Y. Sasajima T. Sumpio B. E. (2007). Effects of different types of fluid shear stress on endothelial cell proliferation and survival. J. Cell. Physiol. 212, 244–251. 10.1002/jcp.21024 17323381

[B43] Kendall R. T. Feghali-Bostwick C. A. (2014). Fibroblasts in fibrosis: Novel roles and mediators. Front. Pharmacol. 5, 123. 10.3389/fphar.2014.00123 24904424 PMC4034148

[B44] Kim K. K. Sheppard D. Chapman H. A. (2018). TGF-β1 signaling and tissue fibrosis. Csh Perspect. Biol. 10, a022293. 10.1101/cshperspect.a022293 PMC5880172 28432134

[B45] Kirkland G. Paizis K. Wu L. L. Katerelos M. Power D. A. (1998). Heparin-binding EGF-like growth factor mRNA is upregulated in the peri-infarct region of the remnant kidney model: *In vitro* evidence suggests a regulatory role in myofibroblast transformation. J. Am. Soc. Nephrol. 9, 1464–1473. 10.1681/asn.v981464 9697669

[B46] Krahn K. N. Bouten C. V. C. Tuijl S. V Zandvoort M. A. M. J. V. Merkx M. (2006). Fluorescently labeled collagen binding proteins allow specific visualization of collagen in tissues and live cell culture. Anal. Biochem. 350, 177–185. 10.1016/j.ab.2006.01.013 16476406

[B47] Kriehuber E. Breiteneder-Geleff S. Groeger M. Soleiman A. Schoppmann S. F. Stingl G. (2001). Isolation and characterization of dermal lymphatic and blood endothelial cells reveal stable and functionally specialized cell lineages. J. Exp. Med. 194, 797–808. 10.1084/jem.194.6.797 11560995 PMC2195953

[B48] Lei X. Liu B. Wu H. Wu X. Wang X. L. Song Y. (2020). The effect of fluid shear stress on fibroblasts and stem cells on plane and groove topographies. Cell. Adhes. Migr. 14, 12–23. 10.1080/19336918.2020.1713532 PMC6973306 31942821

[B49] Lendahl U. Muhl L. Betsholtz C. (2022). Identification, discrimination and heterogeneity of fibroblasts. Nat. Commun. 13, 3409. 10.1038/s41467-022-30633-9 35701396 PMC9192344

[B50] Liberzon A. Birger C. Thorvaldsdóttir H. Ghandi M. Mesirov J. P. Tamayo P. (2015). The Molecular Signatures Database (MSigDB) hallmark gene set collection. Cell. Syst. 1, 417–425. 10.1016/j.cels.2015.12.004 26771021 PMC4707969

[B51] Lin S. L. Kisseleva T. Brenner D. A. Duffield J. S. (2008). Pericytes and perivascular fibroblasts are the primary source of collagen-producing cells in obstructive fibrosis of the kidney. Am. J. Pathol. 173, 1617–1627. 10.2353/ajpath.2008.080433 19008372 PMC2626374

[B52] Liu L. You Z. Yu H. Zhou L. Zhao H. Yan X. (2017). Mechanotransduction-modulated fibrotic microniches reveal the contribution of angiogenesis in liver fibrosis. Nat. Mater 16, 1252–1261. 10.1038/nmat5024 29170554

[B53] Livak K. J. Schmittgen T. D. (2001). Analysis of relative gene expression data using real-time quantitative PCR and the 2(-Delta Delta C(T)) Method. Methods 25, 402–408. 10.1006/meth.2001.1262 11846609

[B54] Lun A. T. L. McCarthy D. J. Marioni J. C. (2016). A step-by-step workflow for low-level analysis of single-cell RNA-seq data with Bioconductor. F1000research 5, 2122. 10.12688/f1000research.9501.2 27909575 PMC5112579

[B55] Malek A. M. Izumo S. (1996). Mechanism of endothelial cell shape change and cytoskeletal remodeling in response to fluid shear stress. J. Cell. Sci. 109, 713–726. 10.1242/jcs.109.4.713 8718663

[B56] Martinez F. J. Collard H. R. Pardo A. Raghu G. Richeldi L. Selman M. (2017). Idiopathic pulmonary fibrosis. Nat. Rev. Dis. Prim. 3, 17074. 10.1038/nrdp.2017.74 29052582

[B57] Mastikhina O. Moon B. U. Williams K. Hatkar R. Gustafson D. Mourad O. (2020). Human cardiac fibrosis-on-a-chip model recapitulates disease hallmarks and can serve as a platform for drug testing. Biomaterials 233, 119741. 10.1016/j.biomaterials.2019.119741 31927251

[B58] McInnes L. Healy J. Melville J. (2018). Umap: Uniform manifold approximation and projection for dimension reduction. Arxiv. 10.48550/arxiv.1802.03426

[B59] Melsted P. Ntranos V. Pachter L. (2019). The barcode, UMI, set format and BUStools. Bioinformatics 35, 4472–4473. 10.1093/bioinformatics/btz279 31073610

[B60] Méndez-Barbero N. Gutiérrez-Muñoz C. Colio L. M. B. (2021). Cellular crosstalk between endothelial and smooth muscle cells in vascular wall remodeling. Int. J. Mol. Sci. 22, 7284. 10.3390/ijms22147284 34298897 PMC8306829

[B61] Moore B. B. Coffey M. J. Christensen P. Sitterding S. Ngan R. Wilke C. A. (2000). GM-CSF regulates bleomycin-induced pulmonary fibrosis via a prostaglandin-dependent mechanism. J. Immunol. 165, 4032–4039. 10.4049/jimmunol.165.7.4032 11034414

[B62] Nakao S. Hafezi-Moghadam A. Ishibashi T. (2012). Lymphatics and lymphangiogenesis in the eye. J. Ophthalmol. 2012, 783163. 10.1155/2012/783163 22523652 PMC3317234

[B63] Noria S. Cowan D. B. Gotlieb A. I. Langille B. L. (1999). Transient and steady-state effects of shear stress on endothelial cell adherens junctions. Circ. Res. 85, 504–514. 10.1161/01.res.85.6.504 10488053

[B64] O’Reilly S. Hugle T. Griffiths B. Krippner A. Laar J. M. V. (2012). T cell derived IL-6 and IL-13 drive fibroblast fibrosis: Implications for systemic sclerosis. Ann. Rheum. Dis. 71, 46–47. 10.1136/annrheumdis-2011-201235.11

[B65] Padmanabhan J. Maan Z. N. Kwon S. H. Kosaraju R. Bonham C. A. Gurtner G. C. (2019). *In vivo* models for the study of fibrosis. Adv. Wound Care 8, 645–654. 10.1089/wound.2018.0909 PMC6904938 31827979

[B66] Park S. Kim J. W. Kim J. H. Lim C. W. Kim B. (2015). Differential roles of angiogenesis in the induction of fibrogenesis and the resolution of fibrosis in liver. Biol. Pharm. Bull. 38, 980–985. 10.1248/bpb.b15-00325 26133707

[B67] Patel A. P. Tirosh I. Trombetta J. J. Shalek A. K. Gillespie S. M. Wakimoto H. (2014). Single-cell RNA-seq highlights intratumoral heterogeneity in primary glioblastoma. Science 344, 1396–1401. 10.1126/science.1254257 24925914 PMC4123637

[B68] Pincha N. Hajam E. Y. Badarinath K. Batta S. P. R. Masudi T. Dey R. (2018). PAI1 mediates fibroblast–mast cell interactions in skin fibrosis. J. Clin. Invest. 128, 1807–1819. 10.1172/jci99088 29584619 PMC5919880

[B69] Poisson J. Lemoinne S. Boulanger C. Durand F. Moreau R. Valla D. (2017). Liver sinusoidal endothelial cells: Physiology and role in liver diseases. J. Hepatol. 66, 212–227. 10.1016/j.jhep.2016.07.009 27423426

[B70] Prestigiacomo V. Weston A. Messner S. Lampart F. Suter-Dick L. (2017). Pro-fibrotic compounds induce stellate cell activation, ECM-remodelling and Nrf2 activation in a human 3D-multicellular model of liver fibrosis. Plos One 12, 0179995. 10.1371/journal.pone.0179995 PMC5493342 28665955

[B71] Ramachandran P. Dobie R. Wilson-Kanamori J. R. Dora E. F. Henderson B. E. P. Luu N. T. (2019). Resolving the fibrotic niche of human liver cirrhosis at single-cell level. Nature 575, 512–518. 10.1038/s41586-019-1631-3 31597160 PMC6876711

[B72] Ramachandran P. Matchett K. P. Dobie R. Wilson-Kanamori J. R. Henderson N. C. (2020). Single-cell technologies in hepatology: New insights into liver biology and disease pathogenesis. Nat. Rev. Gastroentero 17, 457–472. 10.1038/s41575-020-0304-x 32483353

[B73] Rogers M. T. Gard A. L. Gaibler R. Mulhern T. J. Strelnikov R. Azizgolshani H. (2021). A high-throughput microfluidic bilayer co-culture platform to study endothelial-pericyte interactions. Sci. Rep-uk 11, 12225. 10.1038/s41598-021-90833-z PMC8190127 34108507

[B74] Ruze A. Zhao Y. Li H. Gulireba X. Li J. Lei D. (2018). Low shear stress upregulates the expression of fractalkine through the activation of mitogen-activated protein kinases in endothelial cells. Blood Coagul. Fibrin 29, 361–368. 10.1097/mbc.0000000000000701 PMC5965924 29406386

[B75] Sakai N. Tager A. M. (2013). Fibrosis of two: Epithelial cell-fibroblast interactions in pulmonary fibrosis. Biochimica Biophysica Acta Bba - Mol Basis Dis 1832, 911–921. 10.1016/j.bbadis.2013.03.001 PMC4041487 23499992

[B76] Sato Y. Okada F. Abe M. Seguchi T. Kuwano M. Sato S. (1993). The mechanism for the activation of latent TGF-beta during co-culture of endothelial cells and smooth muscle cells: Cell-type specific targeting of latent TGF-beta to smooth muscle cells. J. Cell. Biol. 123, 1249–1254. 10.1083/jcb.123.5.1249 8245129 PMC2119883

[B77] Schrimpf C. Duffield J. S. (2011). Mechanisms of fibrosis: The role of the pericyte. Curr. Opin. Nephrol. Hy 20, 297–305. 10.1097/mnh.0b013e328344c3d4 21422927

[B78] Slegtenhorst B. R. Ramirez O. R. F. Zhang Y. Dhanerawala Z. Tullius S. G. García-Cardeña G. (2018). A mechano-activated cell reporter system as a proxy for flow-dependent endothelial atheroprotection. Slas Discov. 23, 869–876. 10.1177/2472555218761101 29498892

[B79] Smith J. D. Bryant S. R. Couper L. L. Vary C. P. Gotwals P. J. Koteliansky V. E. (1999). Soluble transforming growth factor-beta type II receptor inhibits negative remodeling, fibroblast transdifferentiation, and intimal lesion formation but not endothelial growth. Circ. Res. 84 (10), 1212–1222. 10.1161/01.res.84.10.1212 10347096

[B80] Subramanian A. Tamayo P. Mootha V. K. Mukherjee S. Ebert B. L. Gillette M. A. (2005). Gene set enrichment analysis: A knowledge-based approach for interpreting genome-wide expression profiles. Proc. Natl. Acad. Sci. 102, 15545–15550. 10.1073/pnas.0506580102 16199517 PMC1239896

[B81] Swain S. M. Romac J. M. J. Vigna S. R. Liddle R. A. (2022). Piezo1-mediated stellate cell activation causes pressure-induced pancreatic fibrosis in mice. Jci Insight 7, 158288. 10.1172/jci.insight.158288 PMC9089793 35451372

[B82] Tan K. Keegan P. Rogers M. Lu M. Gosset J. R. Charest J. (2019). A high-throughput microfluidic microphysiological system (PREDICT-96) to recapitulate hepatocyte function in dynamic, re-circulating flow conditions. Lab. Chip 19, 1556–1566. 10.1039/c8lc01262h 30855604

[B83] Topper J. N. Jr M. A. G. (1999). Blood flow and vascular gene expression: Fluid shear stress as a modulator of endothelial phenotype. Mol. Med. Today 5, 40–46. 10.1016/s1357-4310(98)01372-0 10088131

[B84] Traag V. A. Waltman L. Eck N. J. V. (2019). From louvain to leiden: Guaranteeing well-connected communities. Sci. Rep-uk 9, 5233. 10.1038/s41598-019-41695-z PMC6435756 30914743

[B85] Tzima E. Irani-Tehrani M. Kiosses W. B. Dejana E. Schultz D. A. Engelhardt B. (2005). A mechanosensory complex that mediates the endothelial cell response to fluid shear stress. Nature 437, 426–431. 10.1038/nature03952 16163360

[B86] Wang L. X. Yang X. Yue Y. Fan T. Hou J. Chen G. X. (2017). Imatinib attenuates cardiac fibrosis by inhibiting platelet-derived growth factor receptors activation in isoproterenol induced model. Plos One 12, 0178619. 10.1371/journal.pone.0178619 PMC5453565 28570599

[B87] Wei K. Korsunsky I. Marshall J. L. Gao A. Watts G. F. M. Major T. (2020). Notch signalling drives synovial fibroblast identity and arthritis pathology. Nature 582, 259–264. 10.1038/s41586-020-2222-z 32499639 PMC7841716

[B88] Woodfin A. Voisin M. B. Nourshargh S. (2007). PECAM-1: A multi-functional molecule in inflammation and vascular biology. Arterioscler. Thromb. Vasc. Biol. 27, 2514–2523. 10.1161/atvbaha.107.151456 17872453

[B89] Wu C. L. Yin R. Wang S. N. Ying R. (2021). A review of CXCL1 in cardiac fibrosis. Front. Cardiovasc Med. 8, 674498. 10.3389/fcvm.2021.674498 33996954 PMC8113392

[B90] Wu C. F. Chiang W. C. Lai C. F. Chang F. C. Chen Y. T. Chou Y. H. (2013). Transforming growth factor β-1 stimulates profibrotic epithelial signaling to activate pericyte-myofibroblast transition in obstructive kidney fibrosis. Am. J. Pathol. 182, 118–131. 10.1016/j.ajpath.2012.09.009 23142380 PMC3538028

[B91] Wynn T. A. (2011). Integrating mechanisms of pulmonary fibrosis. J. Exp. Med. 208, 1339–1350. 10.1084/jem.20110551 21727191 PMC3136685

[B92] Wynn T. Barron L. (2010). Macrophages: Master regulators of inflammation and fibrosis. Semin. Liver Dis. 30, 245–257. 10.1055/s-0030-1255354 20665377 PMC2924662

[B93] Xie X. Wang F. Zhu L. Yang H. Pan D. Liu Y. (2020). Low shear stress induces endothelial cell apoptosis and monocyte adhesion by upregulating PECAM-1 expression. Mol. Med. Rep. 21, 2580–2588. 10.3892/mmr.2020.11060 32323830 PMC7185273

[B94] Ytrehus K. Hulot J. S. Perrino C. Schiattarella G. G. Madonna R. (2018). Perivascular fibrosis and the microvasculature of the heart. Still hidden secrets of pathophysiology? Vasc. Pharmacol. 107, 78–83. 10.1016/j.vph.2018.04.007 29709645

[B95] Yu H. Zeng Y. Hu J. Li C. (2002). Fluid shear stress induces the secretion of monocyte chemoattractant protein-1 in cultured human umbilical vein endothelial cells. Clin. Hemorheol. Micro 26, 199–207. 12082252

[B96] Zhao F. Cheng T. Yang L. Huang Y. Li C. Han J. (2020). G-CSF inhibits pulmonary fibrosis by promoting BMSC homing to the lungs via SDF-1/CXCR4 chemotaxis. Sci. Rep-uk 10, 10515. 10.1038/s41598-020-65580-2 PMC7324625 32601321

[B97] Zhao M. Wang L. Wang M. Zhou S. Lu Y. Cui H. (2022). Targeting fibrosis: Mechanisms and clinical trials. Signal Transduct. Target Ther. 7, 206. 10.1038/s41392-022-01070-3 35773269 PMC9247101

