# Supplementary material for: Fibroblast activation in response to TGFβ1 is modulated by co-culture with endothelial cells in a vascular organ-on-chip platform
Source: Front Mol Biosci. 2023 Jul 28;10:1160851. doi: 10.3389/fmolb.2023.1160851 (PMC10421749; doi:10.3389/fmolb.2023.1160851)
Supplement: Supplementary file 1 [file DataSheet1.pdf]

Supplementary Material

Fibroblast activation in response to TGFβ1 is modulated by co-culture with endothelial cells in a vascular organ-on-chip platform

Rebeccah Luu[1†], B. Christopher Hoefler[1,a†], Ashley L. Gard[1], Casey R. Ritenour[2], Miles T. Rogers[1,b], Ernest S. Kim[1], Jonathan R. Coppeta[1], Brian P. Cain[1], Brett C. Isenberg[1], Hesham Azizgolshani[1], Oscar R. Fajardo-Ramirez[3], Guillermo García-Cardeña [3], Matthew P. Lech[4], Lindsay Tomlinson[4], Joseph L. Charest[1], Corin Williams[1*]

*Correspondence: Corin Williams, cwilliams@draper.com

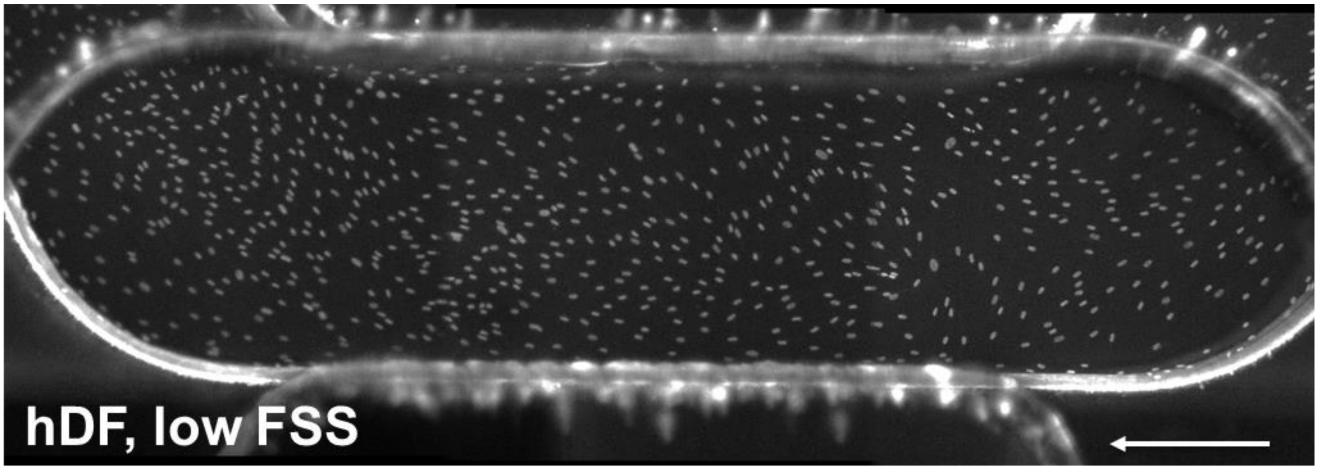

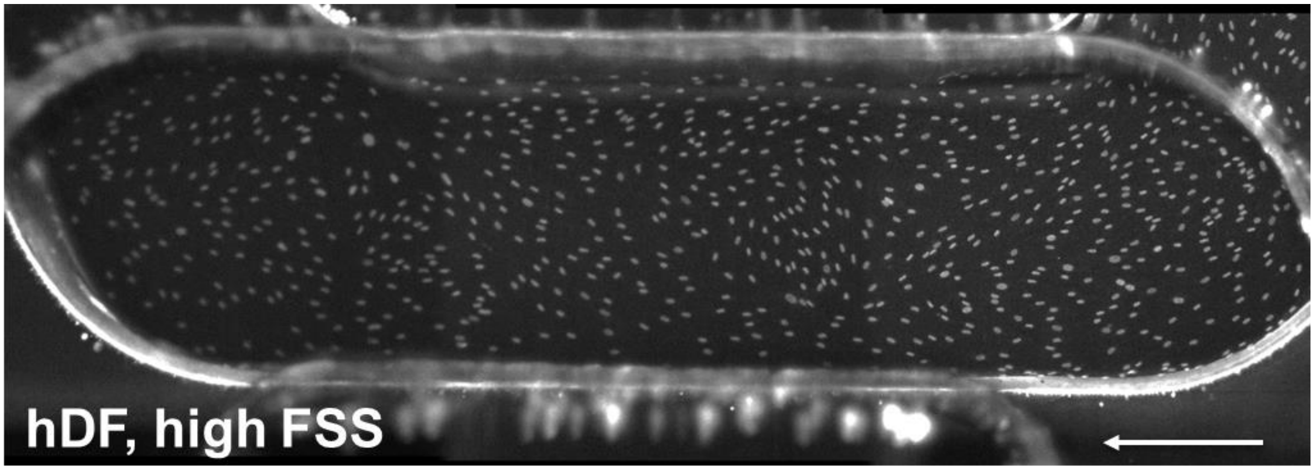

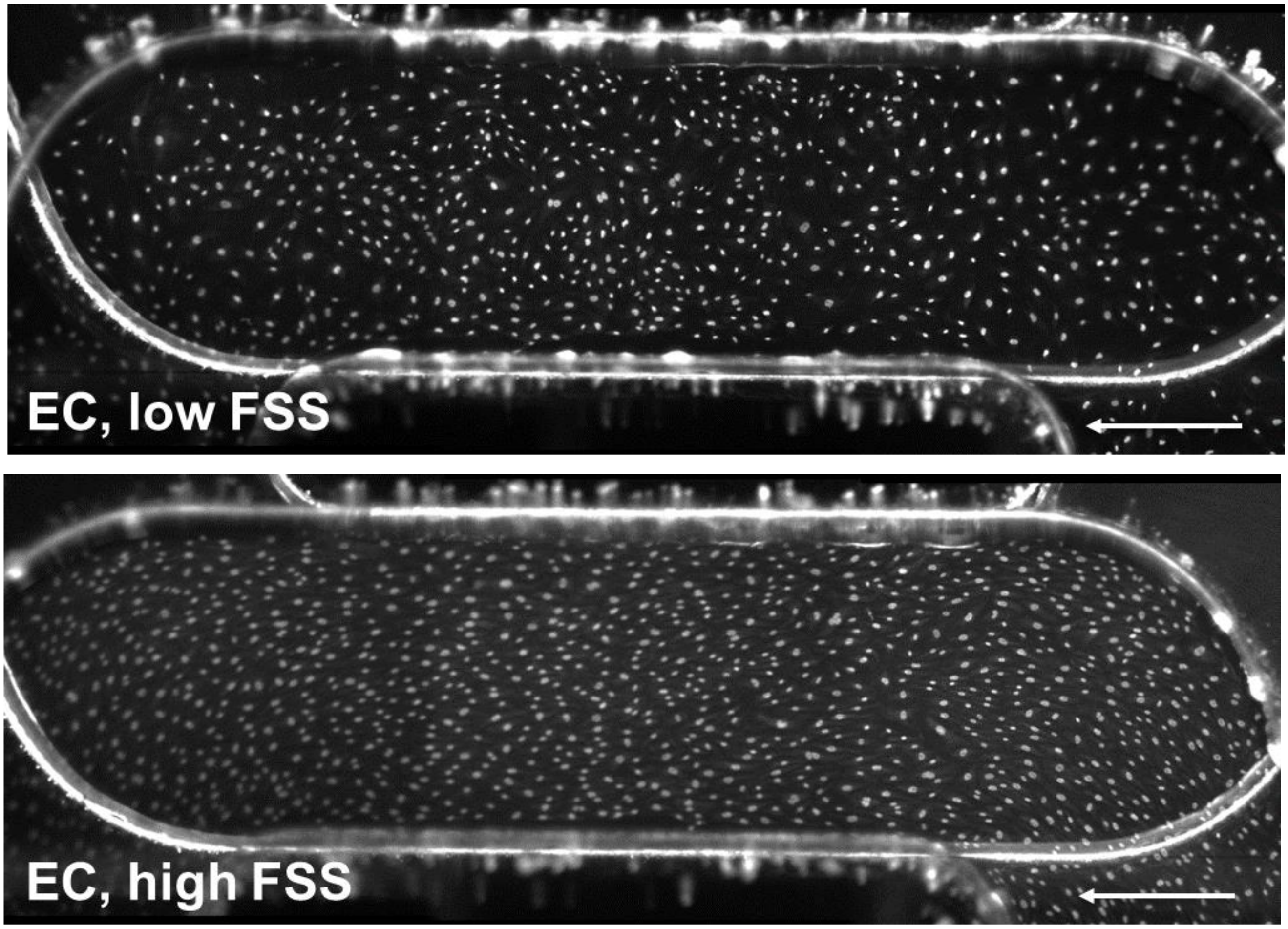

Supplementary Figure 1: Cell nuclei under low and high FSS. Representative images of each cell type under low and high FSS conditions, without TGFβ1. hDF, which are not directly exposed to FSS, do not show any qualitative changes in cell nuclei orientation. EC exposed to low FSS show disorganized nuclei, while EC under high FSS show more uniform coverage and nuclei size with orientation towards flow. Arrows in the bottom right indicate the direction of flow in the bottom channel.

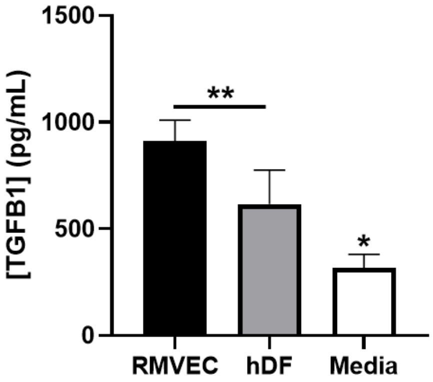

Supplementary Figure 2: TGFβ1 production by EC and hDF. Endogenous levels of TGFβ1 from EC, hDF, and blank co-culture media was determined by ELISA. Baseline media levels were significantly lower than both cell types. One-way ANOVA with Tukey's post-hoc test was used, N = 8 per condition. * $p < 0.001$; ** $p < 0.0001$

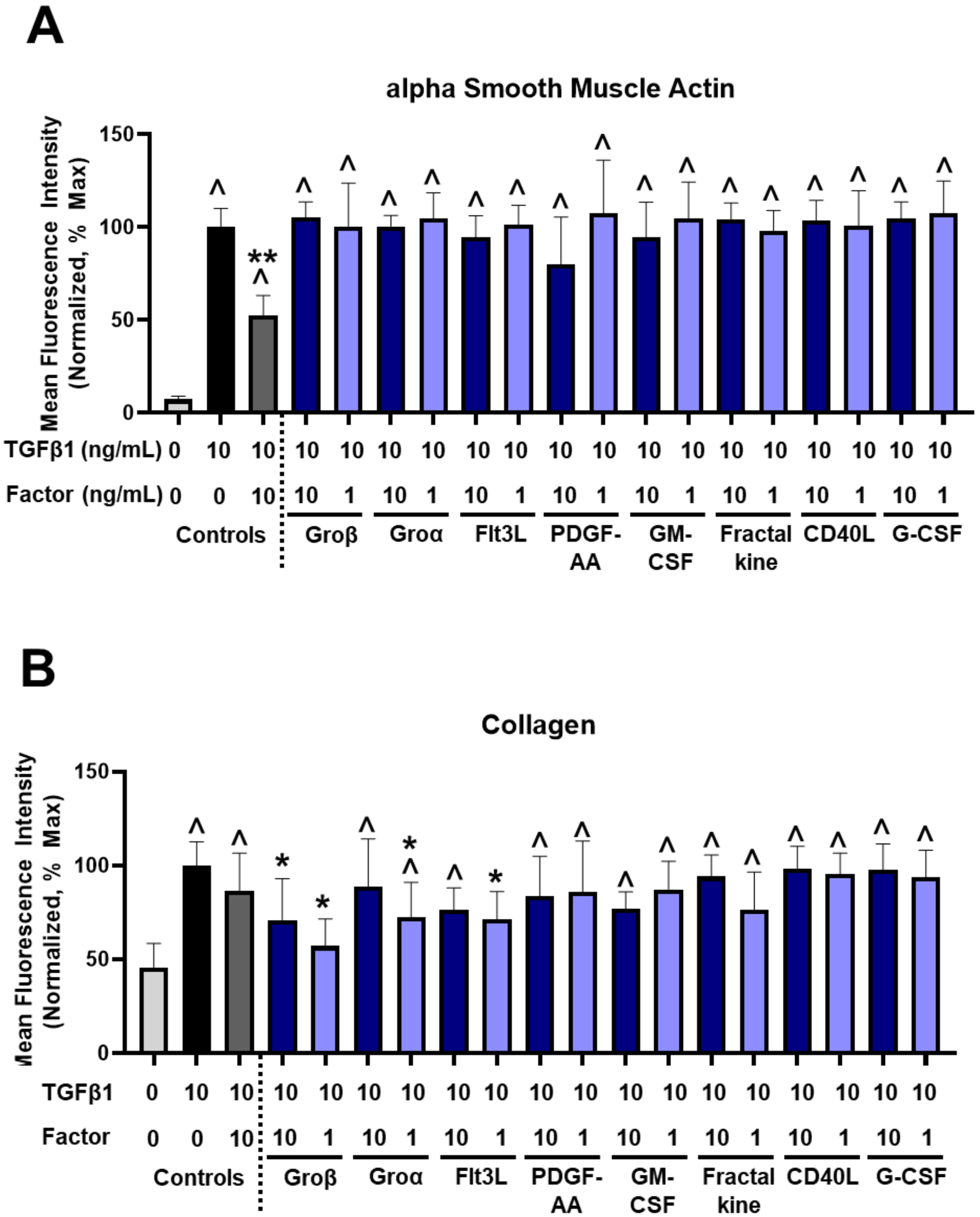

Supplementary Figure 3: The effect of growth factors and cytokines on hDF activation. Various factors that were identified in EC conditioned media were tested with hDF mono-cultures stimulated with TGFβ1. The mean fluorescence intensity of (A) SMA and (B) collagen were measured and normalized to the TGFβ1-treated control condition (assumed to be 100%). One-way ANOVA with Tukey's posthoc test was used for statistical analysis, N = 6-7 samples per condition across two independent experiments. ^ $p < 0.05$ relative to untreated control; * $p < 0.05$ with respect to TGFβ1-treated control; ** $p < 0.0001$ with respect to TGFβ1-treated control.

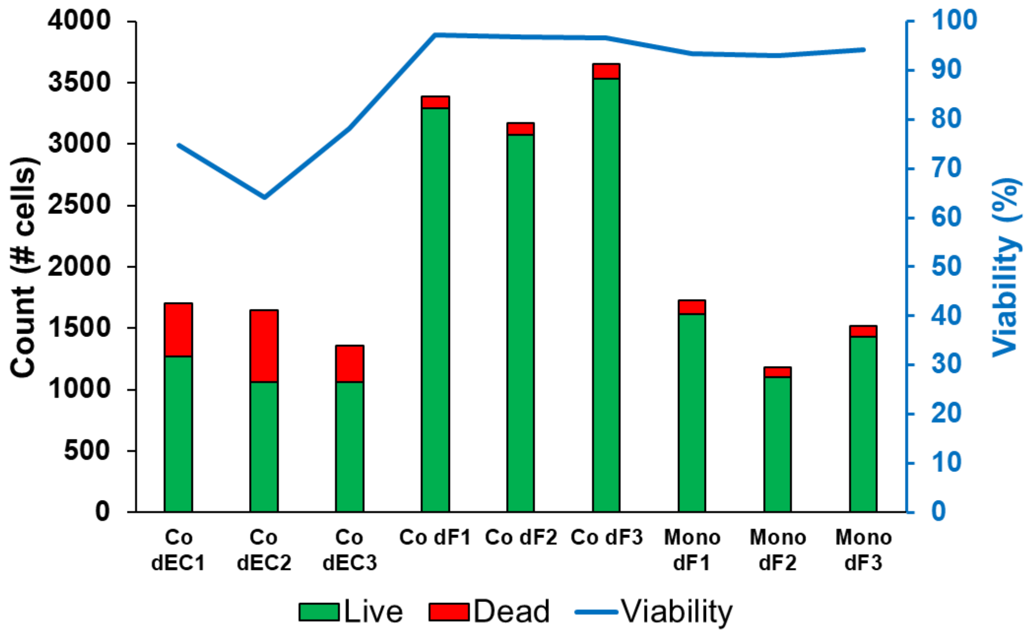

Supplementary Figure 4: Cell numbers and viability for single cell RNA-sequencing. Cell count (left y-axis) and viability (right y-axis, blue) were determined by flow cytometry. Live/dead numbers (green and red bars) were determined by acridine orange and propidium iodide and used to calculate the percentage of cells that were viable (blue line).

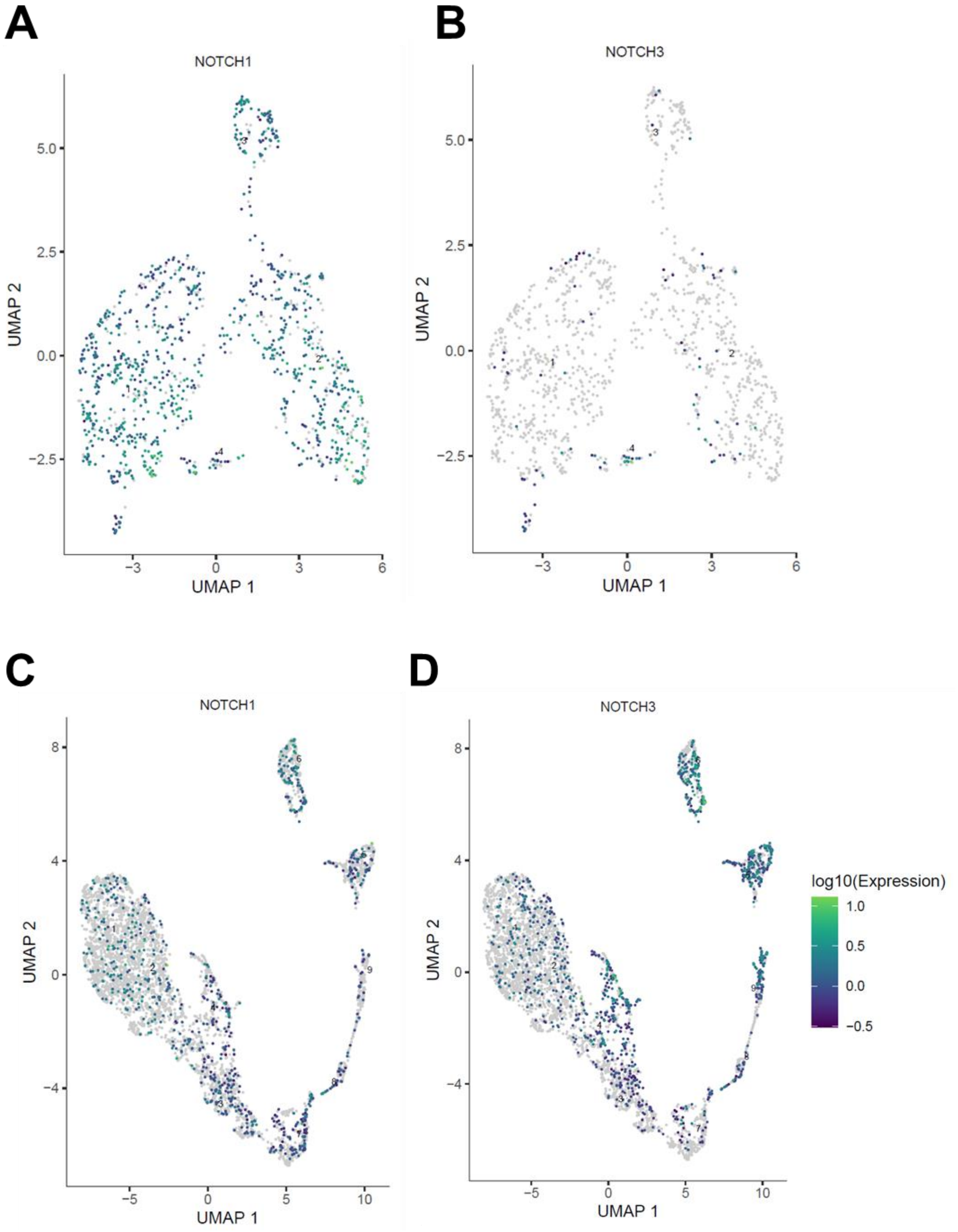

Supplementary Figure 5: Selective Notch receptor expression within cell subtypes by scRNA-seq. (A) EC express NOTCH1 at high levels and (B) NOTCH3 at low to non-existent levels. While some NOTCH1 expression is observed in (C) hDF among the matrix-producing populations, (D) NOTCH3 is highly expressed by most hDF, including proliferating, activated, and matrix-producing phenotypes.

Supplementary Table 1: Growth Factors and Cytokines Used

Factor (Alternative Names)	*Vendor*	*Catalog #*	*Vehicle*
Groα (CXCL1/KC/CINC-1)	R&D Systems	275-GR-010/CF	PBS
CD40 Ligand (TNFSF5)	R&D Systems	6420-CL-025/CF	PBS
Fractalkine (CX3CL1)	R&D Systems	365-FR-025/CF	PBS
Groβ (CXCL2/MIP-2/CINC-3)	R&D Systems	276-GB-010/CF	PBS
G-CSF	R&D Systems	214-CS-005/CF	10 mM acetic acid
PDGF-AA	R&D Systems	221-AA-010	4 mM HCl
Flt-3 Ligand	R&D Systems	308-FKE-010	PBS
GM-CSF	R&D Systems	7954-GM-010/CF	PBS
FGF-2	PeproTech	108-18B	PBS
TGFβ1	R&D Systems	7754-BH-005/CF	0.5% BSA in 4 mM HCl

Supplementary Table 2: Intra- and Inter-plate Variability

SMA MFI	*Mono-culture (-)*	*Mono-culture (+)*	*Co-culture (-)*	*Co-culture (+)*
Intra-plate variability (1)	3.1%	9%	3.4%	9.6%
Intra-plate variability (2)	32%	17.5%	8.2%	18.8%
Intra-plate variability (3)	19%	7.1%	2.5%	6.2%
Inter-plate variability (avg)	18%	11.2%	4.7%	11.5%

Intra-plate coefficient of variance (CV) was calculated for technical replicates within the plate (N=3-4) for each of 3 independent PREDICT96 plates. Inter-plate variability was calculated by averaging the CV values across the 3 plates for a given condition.
